# Evaluation of Interventions to Reduce Opioid Prescribing for Patients Discharged From the Emergency Department: A Systematic Review and Meta-analysis

**DOI:** 10.1001/jamanetworkopen.2021.43425

**Published:** 2022-01-13

**Authors:** Raoul Daoust, Jean Paquet, Martin Marquis, Jean-Marc Chauny, David Williamson, Vérilibe Huard, Caroline Arbour, Marcel Émond, Alexis Cournoyer

**Affiliations:** 1 Study Center in Emergency Medicine, Hôpital du Sacré-Coeur de Montréal, Le Centre Intégré Universitaire de Santé et de Services Sociaux (CIUSSS) du Nord-de-l’Île de-Montréal, Montréal, Québec, Canada; 2 Département de Médecine Familiale et de Médecine d’Urgence, Faculté de Médecine, Université de Montréal, Montréal, Québec, Canada; 3 Centre de Recherche de l’Hôpital du Sacré-Coeur de Montréal, CIUSSS du Nord de-l’Île-de-Montréal, Montréal, Québec, Canada; 4 Faculté de Pharmacie, Université de Montréal, Montréal, Québec, Canada; 5 Faculté des Sciences Infirmières, Université de Montréal, Montréal, Québec, Canada; 6 Département de Médecine Familiale et de Médecine d’Urgence, Faculté de Médecine, Université Laval, Québec, Québec, Canada

## Abstract

**Importance:**

Limiting opioid overprescribing in the emergency department (ED) may be associated with decreases in diversion and misuse.

**Objective:**

To review and analyze interventions designed to reduce the rate of opioid prescriptions or the quantity prescribed for pain in adults discharged from the ED.

**Data Sources:**

MEDLINE, Embase, CINAHL, PsycINFO, and Cochrane Controlled Register of Trials databases and the gray literature were searched from inception to May 15, 2020, with an updated search performed March 6, 2021.

**Study Selection:**

Intervention studies aimed at reducing opioid prescribing at ED discharge were first screened using titles and abstracts. The full text of the remaining citations was then evaluated against inclusion and exclusion criteria by 2 independent reviewers.

**Data Extraction and Synthesis:**

Data were extracted independently by 2 reviewers who also assessed the risk of bias. Authors were contacted for missing data. The main meta-analysis was accompanied by intervention category subgroup analyses. All meta-analyses used random-effects models, and heterogeneity was quantified using *I*^2^ values.

**Main Outcomes and Measures:**

The primary outcome was the variation in opioid prescription rate and/or prescribed quantity associated with the interventions. Effect sizes were computed separately for interrupted time series (ITS) studies.

**Results:**

Sixty-three unique studies were included in the review, and 45 studies had sufficient data to be included in the meta-analysis. A statistically significant reduction in the opioid prescription rate was observed for both ITS (6-month step change, −22.61%; 95% CI, −30.70% to −14.52%) and other (odds ratio, 0.56; 95% CI, 0.45-0.70) study designs. No statistically significant reduction in prescribed opioid quantities was observed for ITS studies (6-month step change, −8.64%; 95% CI, −17.48% to 0.20%), but a small, statistically significant reduction was observed for other study designs (standardized mean difference, −0.30; 95% CI, −0.51 to −0.09). For ITS studies, education, policies, and guideline interventions (6-month step change, −33.31%; 95% CI, −39.67% to −26.94%) were better at reducing the opioid prescription rate compared with prescription drug monitoring programs and laws (6-month step change, −11.18%; 95% CI, −22.34% to −0.03%). Most intervention categories did not reduce prescribed opioid quantities. Insufficient data were available on patient-centered outcomes such as pain relief or patients’ satisfaction.

**Conclusions and Relevance:**

This systematic review and meta-analysis found that most interventions reduced the opioid prescription rate but not the prescribed opioid quantity for ED-discharged patients. More studies on patient-centered outcomes and using novel approaches to reduce the opioid quantity per prescription are needed.

**Trial Registration:**

PROSPERO Identifier: CRD42020187251

## Key Points

**Question:**

Are interventions to reduce pain-related opioid prescribing for patients who are discharged from the emergency department associated with variation in opioid prescription rate and/or prescribed opioid quantity?

**Findings:**

In this meta-analysis of 63 unique studies, 34 of 51 studies assessing prescribing rates (67%) reported a significant reduction after intervention implementation, and 17 of the 39 studies assessing prescribed opioid quantity (44%) reported a significant intervention-related reduction. There were no data on patient-centered outcomes such as pain relief.

**Meaning:**

This analysis found that specific interventions may be associated with reducing the opioid prescription rate; however, novel interventions are needed to reduce the quantity of opioids per prescription by emergency department physicians while evaluating their associations with patient-centered outcomes.

## Teaser

This meta-analysis reviews and analyzes the evidence regarding interventions to reduce the opioid prescribing rate or quantity for treating pain in patients discharged from the emergency department.

## Introduction

Rates of prescription opioid–related deaths have remained very high during the last 10 years in the US.^1^ Despite this ongoing crisis, opioids are still frequently prescribed for home pain management by physicians^2,3,4^ because some patients may need them for pain control when alternatives are not sufficient.^5^ Although a decrease in opioid prescribing has been observed in recent years from emergency department (ED) physicians,^6,7,8^ it can still be optimized because variability in opioid prescribing among ED physicians remains substantial, and overprescribing continues to be frequent.^9,10,11,12,13^ Opioid prescription to patients with acute pain after an ED visit can lead to long-term use^14,15^ and opioid use disorders.^16^ Furthermore, it has been shown that as much as 68% of the initial opioid prescription after an ED visit for acute pain is left unused.^17,18^ These extra pills can be diverted, leading to opioid misuse, dependence, and overdose in our communities.^19,20,21^

Limiting overprescribing could have a substantial effect on opioid diversion and misuse and therefore on the opioid crisis.^22,23^ Several opioid reduction strategies have been proposed by medical institutions, cities, states, and other stakeholders. These approaches vary extensively, ranging from prescription drug monitoring programs,^24,25,26,27^ new laws,^28,29,30^ policies, guidelines,^31,32,33,34^ prescriber education initiatives,^35,36^ or changing the default quantity of opioids in electronic medical record prescription orders.^37,38,39,40^ The efficacy of these interventions on the opioid prescribing rate or quantity at ED discharge remains uncertain and, to our knowledge, has never been examined systematically. The identification of approaches associated with greater efficacy could help policy makers elaborate more targeted programs to prevent opioid misuse and deaths. Our main objective was to review and analyze the evidence regarding interventions to reduce the opioid prescribing rate or quantity for treating pain in adults discharged from the ED.

## Methods

### Review Design

This meta-analysis was registered before its initiation (PROSPERO identifier: CRD42020187251). The results are presented as per the Preferred Reporting Items for Systematic Reviews and Meta-Analysis (PRISMA) reporting guideline (supporting checklist/diagram).^41^ We also followed the Synthesis Without Meta-analysis reporting rules^42^ to complement the PRISMA guideline.

### Eligibility Criteria

We included all intervention studies designed to reduce the opioid prescription rate and/or the quantity of opioids per prescription given to adults discharged from the ED (≥18 years of age) for home pain management. Except for case reports and case series, all types of study designs were included.

Studies that exclusively included pediatric patients or populations with substance use disorder or only evaluated opioids given during the ED stay were excluded. Studies performed in settings other than the ED, that were without interventions, that pertained to opioid use unrelated to pain, or that did not report opioid prescribing rate or quantity were also excluded.

### Data Sources and Search Strategy

In collaboration with an information specialist, we developed a search strategy based on the intersection of 3 search themes: *opioid*, *emergency*, and *prescription* (detailed search strategies are available in eMethods 1 in the Supplement). The following databases were searched from their inception to May 15, 2020: MEDLINE, Embase, CINAHL, PsycINFO, and Cochrane Controlled Register of Trials. An updated search was performed at the end of the process on March 6, 2021, to collect recently published reports. We also searched ClinicalTrials.gov and the International Standard Randomized Controlled Trial Number registry for ongoing studies. Gray literature was searched using Google Scholar, and the first 300 hits were screened to identify any relevant studies. References from studies meeting inclusion criteria were examined to identify additional relevant studies. There were no exclusions based on language.

### Study Selection

The identified references from all databases were transferred to Covidence systematic review manager (Covidence systematic review software, Veritas Health Innovation [https://www.covidence.org]). Six reviewers (R.D., J.P., M.M., J.-M.C., D.W., and A.C.) participated in a 2-stage selection process of eligible studies. After duplicate removal, each citation was screened by 2 independent reviewers using the study title and abstract. Finally, 2 independent reviewers evaluated the full text of the remaining citations against inclusion and exclusion criteria. During both stages, a third reviewer (R.D. or A.C.) resolved discrepancies.

### Data Extraction

The data for all pertinent variables were extracted independently by 2 reviewers (J.P. and M.M.) using a standardized electronic Excel sheet (Microsoft Corporation), and the conflicts were resolved by consensus from the 2 reviewers. Study authors were contacted when outcomes were incomplete or when adult patients in the ED were mixed in with other populations. When the type of pain (acute or chronic) or the problem or diagnosis was not explicitly specified in the studies, we attributed *both* for type of pain and *all* for problem and diagnosis. Also, when time series data could be extracted from the study, we considered it as an interrupted time series (ITS) study; otherwise, we considered it a preintervention-postintervention design, even if the authors reported their study as an ITS.

The Cochrane Effective Practice and Organisation of Care (EPOC) Taxonomy of Implementation Strategies^43^ standard was used to categorize the different interventions into changes in health care organizations, in the clinician’s behaviors, and in the patient’s use of health services. A consensus was obtained across 6 reviewers (R.D., J.P., M.M., J.-M.C., D.W., and A.C.) to categorize the interventions as (1) education, policy, or guidelines (EPG); (2) prescription drug monitoring program or state law (PDMP); (3) clinician peer comparison (CPC); (4) electronic medical record quantity changes (EMR-QC); and (5) physical therapy (PT) (this category was originally planned as “other” but was finally composed of only physical therapy studies). Clinician peer comparison was categorized separately from EPG because of the additional motivational incentive induced by peer comparisons. A more detailed description of each intervention and the manner and rationale for how they were regrouped are presented in eMethods 2 in the Supplement. Each study was classified into 1 of the 5 intervention categories by 2 independent reviewers (R.D. and J.P.), and discrepancies were resolved by a third reviewer (A.C.). Studies using more than 1 intervention category were classified into the predominant category by reviewer consensus.

### Primary and Secondary Outcomes

Our primary outcome was the variation in the opioid prescription rate and/or quantity generated by the intervention. Our secondary outcomes were the patients’ level of pain relief, patients’ satisfaction with their opioid prescription, and percentage of patients requiring additional opioid prescriptions.

### Quality Assessment and Risk of Bias

The quality assessment of all retained articles was performed by 2 independent reviewers (J.P. and M.M.); conflicts were resolved either by consensus or by a third reviewer (R.D.). The risk of bias for preintervention-postintervention and ITS study designs were evaluated using the Risk of Bias in Nonrandomised Studies of Interventions (ROBINS-I) tool.^44^ The risk of bias for the cohort studies and randomized controlled trials were evaluated using the EPOC risk of bias tool.^45^ Abstracts were automatically considered at critical (ROBINS-I) or high (EPOC) risk of bias.

### Data Synthesis and Analysis

Descriptive statistics were performed for intervention categories, study designs, country of origin, and type of pain (acute or chronic). For each study, we presented the absolute reduction in the opioid prescription rates and/or the absolute change in total amount of opioids per prescription. Rates were reported in percentage of discharged adults who were prescribed opioids. Quantities of opioids per prescription were presented as median (IQR) or mean (SD) number of pills or in total milligram morphine equivalent or morphine equivalent daily dosage. We also calculated the proportion of studies that showed a trend or a statistically significant (2-sided *P* < .05) reduction of opioid prescribing rate or quantity.

If the rate or quantity of opioid prescribed was reported in at least 3 studies, the results were pooled and included separately in a meta-analysis for each type of outcome and each type of study design (ITS vs other). For preintervention-postintervention, cohort, and randomized clinical trial (RCT) study designs, the opioid prescription rate was expressed as an odds ratio (OR). The number of events (opioid prescriptions) over the number of patients discharged from the ED for preintervention/control and postintervention/treatment groups was used to compute the OR. Opioid quantities per prescription were expressed as standardized mean difference (SMD). When means and SDs were not available, they were estimated based on medians or other statistics using the methods developed by Wan et al.^46^ Studies without sufficient data for these analyses were only described.

The ITS studies were analyzed according to Cochrane (EPOC) recommendations.^47^ Because ITS data were not examined uniformly across studies, we reanalyzed all available ITS study data using the same method, as recommended by Ramsay et al,^48,49^ and calculated the 6-month step change. Details of our approach to standardize ITS study results are available in eFigure 1 in the Supplement.

The rate and quantity of prescribed opioids are described according to the 5 intervention categories defined previously. All results are reported with 95% CIs. Because of the different natures of ITS effect size compared with the OR and SMD, these forest plots are presented separately. Heterogeneity was assessed statistically both overall and for each intervention category using *I*^2^ values. The τ^2^ and Cochran Q tests for heterogeneity are also reported. A χ^2^ test was used to determine whether there was a difference between intervention categories. When more than 2 intervention categories were statistically significant, pairwise subgroup χ^2^ tests were performed. All analyses were conducted using an inverse variance weighting method and a random-effects model, even if the *I*^2^ value was low, owing to the diversity of interventions.^50^

For each analysis of more than 10 studies, a funnel plot and Egger test^51^ were used to assess small samples publication bias.^52^ In 2 sets of sensitivity analyses, we used the 1-year step change for ITS studies when available and excluded studies at high risk of bias to evaluate differences in effect size. Segmented time-series regression analyses were performed using SPSS, version 26 (IBM Corporation); forest plots were executed using Revman, version 5.4 (The Nordic Cochrane Centre, The Cochrane Collaboration, 2014); and funnel plots and Egger tests were performed using Comprehensive Meta-Analysis program.^53^

## Results

### Search Results and Study Characteristics

**Figure 1.**
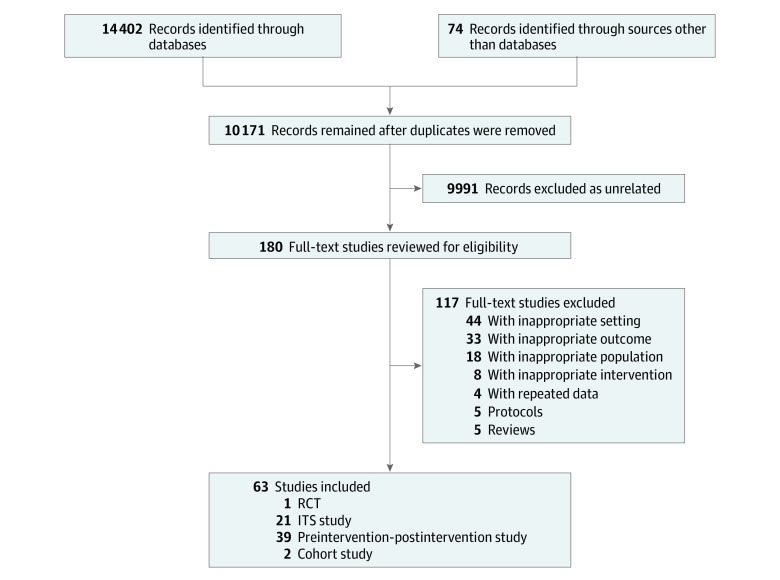
PRISMA Flowchart of Included Studies ITS indicates interrupted time series; RCT, randomized clinical trial.

The initial search strategy generated 10 171 references after duplicate removal. Of these, 180 were kept for full-text review, and 63 unique studies were included in the review (Figure 1).^12,27,28,29,30,31,32,35,36,37,39,40,54,55,56,57,58,59,60,61,62,63,64,65,66,67,68,69,70,71,72,73,74,75,76,77,78,79,80,81,82,83,84,85,86,87,88,89,90,91,92,93,94,95,96,97,98,99,100,101,102,103,104^ Fifteen of 32 authors responded to our requests for more details on outcomes or population, which improved data availability for 10 studies.^12,27,31,36,55,57,67,81,84,86^ Most included studies were conducted in the US (55 [87%]),^12,27,28,29,30,31,32,37,39,54,55,56,57,58,59,60,61,62,63,64,65,66,67,71,72,73,74,75,76,77,78,79,80,81,82,83,84,85,86,87,88,89,90,91,92,93,95,96,97,98,99,100,101,103,104^ 5 were from Australia,^35,36,40,68,70^ and 3 were from Canada.^69,94,102^ All studies were published within the last 10 years (2013 to 2021). Studies used mainly preintervention-postintervention (n = 39)^12,28,30,31,32,35,39,40,58,59,60,61,62,63,64,65,66,67,68,69,70,75,76,77,78,79,80,81,82,83,84,85,92,93,94,99,100,101,102^ and ITS (n = 21)^27,29,36,37,54,55,56,57,71,72,73,74,87,88,89,90,91,95,96,97,98^ designs; only 1 was an RCT,^86^ and 2 were cohort studies.^103,104^ The EPG intervention was used to help reduce opioid prescriptions in 21 studies^31,32,35,36,54,55,56,57,58,59,60,61,62,63,64,65,66,67,68,69,70^; PDMP, in 19 studies^27,28,29,30,71,72,73,74,75,76,77,78,79,80,81,82,83,84,85^; EMR-QC, in 11 studies^37,39,40,95,96,97,98,99,100,101,102^; CPC, in 10 studies^12,86,87,88,89,90,91,92,93,94^; and PT, in 2 studies.^103,104^ Fifty-two studies^12,27,28,29,31,35,36,37,39,40,54,55,57,59,60,61,62,65,66,68,69,70,71,72,73,74,75,76,77,78,79,80,81,83,84,85,86,87,88,89,90,91,92,93,95,96,97,98,99,100,101,102^ included a mix of pain problems, and 56 included both acute and chronic pain.^12,27,28,29,30,31,32,35,36,37,39,40,54,55,56,57,60,61,62,65,66,68,69,70,71,72,73,74,75,76,77,78,79,80,81,84,85,86,87,88,89,90,91,92,93,94,95,96,97,98,99,100,101,102,103,104^ The opioid prescription rate was reported for 25 studies,^28,29,32,54,56,58,59,60,62,63,66,67,69,73,74,75,76,78,79,80,87,89,91,94,104^ the prescribed opioid quantity for 13 studies,^35,37,39,40,68,70,83,93,95,96,97,98,102^ and both for 25 studies^12,27,30,31,36,55,57,61,64,65,71,72,77,81,82,84,85,86,88,90,92,99,100,101,103^ (Table 1).

**Table 1.** Study Characteristics by Intervention Category and Study Design

Study	Intervention category	Study design	Problem/diagnosis^a^	Acute or chronic pain	Intervention description	Primary outcomes reported
Gugelmann et al,^54^ 2013	EPG	ITS	All	Both	Interdisciplinary educational modalities	Rate
Osborn et al,^55^ 2017	EPG	ITS	All	Both	Washington ED opioid prescribing guidelines	Rate and quantity
Beaudoin et al,^56^ 2018	EPG	ITS	Abdominal, dental, back, headache, chronic	Both	ED opioid prescribing policy	Rate
Acquisto et al,^57^ 2019	EPG	ITS	All	Both	Interdisciplinary task force education	Rate and quantity
Kline et al,^36^ 2019	EPG	ITS	All	Both	Education on oxycodone prescription	Rate and quantity
Fox et al,^58^ 2013	EPG	Preintervention-postintervention	Dental pain	Acute	Controlled substance prescribing guidelines	Rate
Jacobs and Vearrier,^59^ 2015	EPG	Preintervention-postintervention	All	Chronic	ED opioid prescription guidelines	Rate
McGhee et al,^60^ 2015	EPG	Preintervention-postintervention	All	Both	Statewide opiate prescribing policy	Rate
del Portal et al,^32^ 2016	EPG	Preintervention-postintervention	Dental, back, neck, chronic	Both	ED opioid prescription guidelines	Rate
Chacko et al,^61^ 2017	EPG	Preintervention-postintervention	All	Both	Opioid prescribing policy	Rate and quantity
Divino et al,^62^ 2017	EPG	Preintervention-postintervention	All	Both	ER/LA risk evaluation and mitigation strategies	Rate
Donaldson et al,^35^ 2017	EPG	Preintervention-postintervention	All	Both	Education ED opioid prescribing	Quantity
Motov et al,^63^ 2018	EPG	Preintervention-postintervention	Renal colic	Acute	Implementation of opioid reduction initiative	Rate
Pace et al,^64^ 2018	EPG	Preintervention-postintervention	Abdominal and back pain	Chronic	Chronic pain management pathway	Rate and quantity
Lowy et al,^65^ 2020	EPG	Preintervention-postintervention	All	Both	Education of interns	Rate and quantity
Dayer et al,^31^ 2019	EPG	Preintervention-postintervention	All	Both	CDC guidelines for opioid prescribing for chronic pain	Rate and quantity
Gordon et al,^66^ 2019	EPG	Preintervention-postintervention	All	Both	Electronic prescribing for controlled substance	Rate
Minhaj et al,^67^ 2020	EPG	Preintervention-postintervention	Renal colic	Acute	Opioid guidelines implementation	Rate
Pattullo et al,^68^ 2020	EPG	Preintervention-postintervention	All	Both	Adaptation of the opioid prescribing toolkit	Quantity
Hartmann et al,^69^ 2021	EPG	Preintervention-postintervention	All	Both	Opioid prescribing guideline	Rate
Pattullo et al,^70^ 2021	EPG	Preintervention-postintervention	All	Both	Oxycodone quality improvement strategy	Quantity
Sun et al,^71^ 2017	PDMP	ITS	All	Both	Best practice mandates on opioid prescription	Rate and quantity
Suffoletto et al,^27^ 2018	PDMP	ITS	All	Both	Effect of PDMP on opioid prescribing	Rate and quantity
Sun et al,^72^ 2018	PDMP	ITS	All	Both	Automated PDMP	Rate and quantity
Bornstein et al,^73^ 2019	PDMP	ITS	All	Both	PDMP implementation and a 2-h course	Rate
Duppong et al,^29^ 2020	PDMP	ITS	All	Both	5-d Supply for acute pain and PDMP	Rate
Liu et al,^74^ 2020	PDMP	ITS	All	Both	Rescheduling of hydrocodone to level II	Rate
McAllister et al,^75^ 2015	PDMP	Preintervention-postintervention	All	Both	Pharmacist provided summary PDMP data	Rate
Jones et al,^76^ 2016	PDMP	Preintervention-postintervention	All	Both	Rescheduling of hydrocodone combination	Rate
Antkowiak et al,^77^ 2018	PDMP	Preintervention-postintervention	All	Both	Controlled substance law effect	Rate and quantity
Love et al,^78^ 2018	PDMP	Preintervention-postintervention	All	Both	State law PDMP evaluation	Rate
Martello et al,^79^ 2018	PDMP	Preintervention-postintervention	All	Both	Education on Pennsylvania PDMP state law	Rate
Khobrani et al,^30^ 2019	PDMP	Preintervention-postintervention	Back pain	Both	State law PDMP evaluation	Rate and quantity
Watson et al,^80^ 2021	PDMP	Preintervention-postintervention	All	Both	Mandated PDMP check of opioid prescription	Rate
Weiner et al,^81^ 2019	PDMP	Preintervention-postintervention	All	Both	1-Click PDMP integration in EMR	Rate and quantity
Young et al,^82^ 2020	PDMP	Preintervention-postintervention	Trauma with minimal injury	Acute	Outpatient opioid prescribing law in Ohio	Rate and quantity
Danovich et al,^28^ 2019	PDMP	Preintervention-postintervention	All	Both	New York State mandate to electronic prescribing of controlled substance	Rate
Perry et al,^83^ 2021	PDMP	Preintervention-postintervention	All	Acute	North Carolina STOP-Act of opioid prescription	Quantity
Sigal et al,^84^ 2021	PDMP	Preintervention-postintervention	All	Both	PDMP and alternative to opioid education	Rate and quantity
Zeiner et al,^85^ 2021	PDMP	Preintervention-postintervention	All	Both	Review PDMP for opioid prescription >72 h	Rate and quantity
Michael et al,^86^ 2018	CPC	RCT	All	Both	Peer comparison of opioid prescription	Rate and quantity
Guarisco and Salup,^87^ 2018	CPC	ITS	All	Both	Sharing of clinician opioid prescription patterns	Rate
Meisenberg et al,^88^ 2018	CPC	ITS	All	Both	Education, peer comparison of opioid prescription	Rate and quantity
Andereck et al,^89^ 2019	CPC	ITS	All	Both	Peer comparison feedback	Rate
Boyle et al,^90^ 2019	CPC	ITS	All	Both	Sharing of clinician opioid prescription patterns	Rate and quantity
Dieujuste et al,^91^ 2020	CPC	ITS	All	Both	ED-based opioid safety initiative	Rate
Burton et al,^12^ 2016	CPC	Preintervention-postintervention	All	Both	Peer comparison feedback	Rate and quantity
Schaefer et al,^92^ 2018	CPC	Preintervention-postintervention	All	Both	Education and feedback to clinicians	Rate and quantity
Anhalt et al,^93^ 2019	CPC	Preintervention-postintervention	All	Both	Peer comparison feedback	Quantity
Yang et al,^94^ 2020	CPC	Preintervention-postintervention	Low back pain	Both	Implementation of clinician performance indicators	Rate
Delgado et al,^37^ 2018	EMR_QC	ITS	All	Both	Default quantity of 10 pills in EMR	Quantity
Slovis et al,^95^ 2018	EMR_QC	ITS	All	Both	Decreasing EMR default opioid prescribing	Quantity
Beauchamp et al,^96^ 2020	EMR_QC	ITS	All	Both	EMR modification to include taper protocol	Quantity
Villwock et al,^97^ 2020	EMR_QC	ITS	All	Both	Removing the 30 pills default from EMR for opioid prescription	Quantity
Carlson et al,^98^ 2020	EMR_QC	ITS	All	Both	Default dispense quantity 17 pills in EMR	Quantity
Zwank et al,^99^ 2017	EMR_QC	Preintervention-postintervention	All	Both	Removing default EMR opioid of 15 pills	Rate and quantity
Santistevan et al,^39^ 2018	EMR_QC	Preintervention-postintervention	All	Both	Removing of default 20 pills of opioid in EMR	Quantity
Schwartz et al,^40^ 2019	EMR_QC	Preintervention-postintervention	All	Both	Including smaller quantity of opioid in EMR default	Quantity
Smalley et al,^100^ 2020	EMR_QC	Preintervention-postintervention	All	Both	EMR opioid default limitations and feedback	Rate and quantity
Johnson et al,^101^ 2020	EMR_QC	Preintervention-postintervention	All	Both	Multidisciplinary opioid reduction package	Rate and quantity
Shelton et al,^102^ 2020	EMR_QC	Preintervention-postintervention	All	Both	Implementation of an electronic prescription writer	Quantity
Kim et al,^103^ 2019	PT	Cohort	Back and neck pain	Both	Initial physical therapist consultation	Rate and quantity
Pugh et al,^104^ 2020	PT	Cohort	Musculoskeletal	Both	Dedicated physical therapy consultation	Rate

Abbreviations: CDC, Centers for Disease Control and Prevention; CPC, clinician peer comparison opioid prescribing; ED, emergency department; EMR, electronic medical record; EMR_QC, EMR quantity changes; EPG, education, policy, or guidelines evaluation; ER, extended release; ITS, interrupted time series; LA, long acting; PDMP, prescription drug monitoring program or state law; PT, physical therapy consultation; RCT, randomized clinical trial; STOP-Act, Strengthen Opioid Misuse Prevention.

^a^ When the type of pain (acute or chronic) or complaints/diagnosis were not explicitly specified in the studies, we selected *all* for problem/diagnosis and *both* for type of pain.

### Risk of Bias Assessment

Ten of 21 ITS studies^27,36,54,55,56,57,88,89,90,91^ demonstrated an overall moderate risk of bias (ROBINS-I); all other ITS and preintervention-postintervention studies^12,28,29,30,31,32,35,37,39,40,58,59,60,61,62,63,64,65,66,67,68,69,70,71,72,73,74,75,76,77,78,79,80,81,82,83,84,85,87,92,93,94,95,96,97,98,99,100,101,102^ were at serious or critical risk (eFigure 2 in the Supplement). The only included RCT^86^ was at low overall risk of bias, and both cohort studies^103,104^ were at high risk of bias (EPOC) (eFigure 3 in the Supplement).

### Primary Descriptive Results

The absolute reduction of the prescribed opioid rate and quantity for included studies is presented in Table 2. Two studies^37,54^ reported and analyzed separately the results from their 2 sites (sites A and B). Of 51 studies assessing prescribing rates, 46 (90%) reported a reduction after intervention implementation (34 were statistically significant).^12,27,28,29,31,32,54,55,56,57,58,59,62,63,64,65,66,67,69,76,77,78,79,80,82,84,85,86,88,90,94,100,101^ Of 39 studies assessing prescribed opioid quantity, 32 (82%) reported an intervention-related reduction (17 were statistically significant).^35,36,39,40,55,65,68,70,82,83,84,85,86,95,98,100,101^

**Table 2.** Primary Outcomes by Intervention Category and Study Design

Source	Intervention category/study design	Rate of opioid prescription					Quantity of opioid per prescription			
		Outcome/unit	Preintervention/control	Postintervention/treatment	Absolute reduction	Outcome/unit		Preintervention/control	Postintervention/treatment	Absolute reduction
Gugelmann et al,^54^ 2013A	EPG/ITS	6-MSC/% of prescription	12.8%	8.0%	−4.8%^a^	NA		NA	NA	NA
Gugelmann et al,^54^ 2013B	EPG/ITS	6-MSC/% of prescription	4.6%	2.3%	−2.3%^a^	NA		NA	NA	NA
Osborn et al,^55^ 2017	EPG/ITS	6-MSC/% of prescription	24.7%	17.2%	−7.5%^a^	6-MSC/No. of pills		20.2	17.5	−2.7^a^
Beaudoin et al,^56^ 2018	EPG/ITS	6-MSC/No. of prescriptions	571.6	410.5	−161.1^a^	NA		NA	NA	NA
Acquisto et al,^57^ 2019	EPG/ITS^b^	6-MSC/No. per 1000	90.7	55.3	−35.4^a^	Mean (SD) MME		113.9 (82.6)	111.7 (83.2)	−2.2
Kline et al,^36^ 2019	EPG/ITS	6-MSC/No. per 1000	32.1	24.3	−7.8	6-MSC/No. of pills		15.8	11.4	−4.4^a^
Fox et al,^58^ 2013	EPG/preintervention-postintervention	Rate/%	59%	42%	−17%^a^	NA		NA	NA	NA
Jacobs and Vearrier,^59^ 2015	EPG/preintervention-postintervention	Rate/%	41%	18%	−23%^a^	NA		NA	NA	NA
McGhee et al,^60^ 2015	EPG/preintervention-postintervention	Mean (SD) prescription per day	405 (4.5)	367 (27.9)	−38	NA		NA	NA	NA
del Portal et al,^32^ 2016	EPG/preintervention-postintervention	Rate/%	52.7%	29.8%	−22.9%^a^	NA		NA	NA	NA
Chacko et al,^61^ 2017	EPG/preintervention-postintervention	No. of prescriptions	1745	1127	−618^c^	Mean MME		69.4	69.0	−0.4^c^
Divino et al,^62^ 2017	EPG/preintervention-postintervention	Quarterly mean	41 449	30 861	−10 588^a^	NA		NA	NA	NA
Donaldson et al,^35^ 2017	EPG/preintervention-postintervention	NA	NA	NA	NA	Median mg oxycodone		100	50	−50^a^
Motov et al,^63^ 2018	EPG/preintervention-postintervention	Rate/%	69%	43%	−26%^a^	NA		NA	NA	NA
Pace et al,^64^ 2018	EPG/preintervention-postintervention	Rate/%	37%	23%	−14%^a^	Mean (SD) MME		132.3 (95.6)	105.8 (76.7)	−26.5
Lowy et al,^65^ 2020	EPG/preintervention-postintervention	Rate/%	2%	0.5%	−1.5%^a^	Mean (SD) MME		37.7 (26.6)	31.8 (15.6)	−5.9^a^
Dayer et al,^31^ 2019	EPG/preintervention-postintervention	Rate/%	16.5%	15.5%	−1%^a^	Mean (SD) MEDD		29.1 (20.4)	28.4 (18.7)	−0.7
Gordon et al,^66^ 2019	EPG/preintervention-postintervention	Rate/%	7.5%	4.8%	−2.7%^a^	NA		NA	NA	NA
Minhaj et al,^67^ 2020	EPG/preintervention-postintervention	Rate/%	69%	61%	−8%^a^	NA		NA	NA	NA
Pattullo et al,^68^ 2020	EPG/preintervention-postintervention	NA	NA	NA	NA	% > 10 Pills		65%	15%	−50%^a^
Hartmann et al,^69^ 2021	EPG/preintervention-postintervention	Mean MME per patient	10.4	7.1	−3.3^a^	NA		NA	NA	NA
Pattullo et al,^70^ 2021	EPG/preintervention-postintervention	NA	NA	NA	NA	% > 10 Pills		38%	10%	−28%^a^
Sun et al,^71^ 2017	PDMP/ITS	6-MSC/% of prescriptions	18.4%	16.7%	−1.7%	6-MSC/MME		188.6	177.5	−11.1
Suffoletto et al,^27^ 2018	PDMP/ITS	6-MSC/% of prescriptions	9.2%	7.1%	−2.1%^a^	6-MSC/% >12 pills		24.4%	26.7%	+2.3%
Sun et al,^72^ 2018	PDMP/ITS	6-MSC/% of prescriptions	19.8%	19.1%	−0.7%	6-MSC/MME		105.8	109.8	+4.0
Bornstein et al,^73^ 2019	PDMP/ITS	6-MSC/% of prescriptions	3.4%	3.2%	−0.18%	NA		NA	NA	NA
Duppong et al,^29^ 2020	PDMP/ITS	6-MSC/No. of prescriptions	1290	805	−485^a^	NA		NA	NA	NA
Liu et al,^74^ 2020	PDMP/ITS	6-MSC/No. of prescriptions	14 662	14 750	+88	NA		NA	NA	NA
McAllister et al,^75^ 2015	PDMP/preintervention-postintervention	Rate/%	19.5%	23.6%	+4.1%	NA		NA	NA	NA
Jones et al,^76^ 2016	PDMP/preintervention-postintervention	Quarterly mean	6 162 419	5 100 292	−1 062 127^a^	NA		NA	NA	NA
Antkowiak et al,^77^ 2018	PDMP/preintervention-postintervention	Mean (SD) prescriptions per month	508 (51)	353 (66)	−155^a^	Mean MME		124.5	112.9	−11.6^c^
Love et al,^78^ 2018	PDMP/preintervention-postintervention	Mean %	5.6%	2.7%	−2.9%^a^	NA		NA	NA	NA
Martello et al,^79^ 2018	PDMP/preintervention-postintervention	Rate/%	21.5%	16.1%	−5.4%^a^	NA		NA	NA	NA
Khobrani et al,^30^ 2019	PDMP/preintervention-postintervention	Rate/%	46%	48%	+2%	Median (IQR) MME		75 (60-120)	75 (60-90)	0
Watson et al,^80^ 2021	PDMP/preintervention-postintervention	Median (IQR) rate per month	0.16 (0.13-0.18)	0.05 (0.04-0.06)	−0.09^a^	NA		NA	NA	NA
Weiner et al,^81^ 2019	PDMP/preintervention-postintervention	No. of prescriptions	5800	5621	−179^c^	Mean MME		137	113	−24^c^
Young et al,^82^ 2020	PDMP/preintervention-postintervention	Rate/%	31%	16%	−15%^a^	Median (IQR) MME		225 (113-338)	90 (60-128)	−135^a^
Danovich et al,^28^ 2019	PDMP/preintervention-postintervention	Rate/%	6.1%	2.9%	−3.2%^a^	NA		NA	NA	NA
Perry et al,^83^ 2021	PDMP/preintervention-postintervention	NA	NA	NA	NA	% >5 d		9.8%	6.5%	−3.3%^a^
Sigal et al,^84^ 2021	PDMP/preintervention-postintervention	Rate/%	19.4%	7.4%	−12%^a^	Mean (SD) MME		104.9 (72.7)	86.8 (110.4)	−18.1^a^
Zeiner et al,^85^ 2021	PDMP/preintervention-postintervention	No. of prescriptions	5798	4509	−1289^a^	Median (IQR) MME		100 (75-120)	90 (60-100)	−10^a^
Michael et al,^86^ 2018	CPC/RCT	Rate/%	14.2%	8.7%	−5.5%^a^	Median (IQR) MME		98 (75-120)	90 (64-117)	−8^a^
Guarisco and Salup,^87^ 2018	CPC/ITS	6-MSC/% of prescriptions	21.6%	17.4%	−4.2%	NA		NA	NA	NA
Meisenberg et al,^88^ 2018	CPC/ITS^b^	6-MSC/MME per visit	21.5	12.4	−9.1^a^	Median (IQR) MME		98 (74-150)	75 (59-90)	−23^c^
Andereck et al,^89^ 2019	CPC/ITS	6-MSC/% of prescriptions	6.5%	5.0%	−1.5%	NA		NA	NA	NA
Boyle et al,^90^ 2019	CPC/ITS^b^	6-MSC/% of prescriptions	16.9%	8.2%	−8.7%^a^	Median (IQR) No. of pills		17 (14-18)	15 (12-16)	−2
Dieujuste et al,^91^ 2020	CPC/ITS	6-MSC/% of prescriptions	7.9%	6.5%	−1.4%	NA		NA	NA	NA
Burton et al,^12^ 2016	CPC/preintervention-postintervention	Rate/%	20.0%	8.3%	−11.7%^a^	Mean (SD) No. of pills		16.7 (4.8)	13.5 (3.3)	−3.2^c^
Schaefer et al,^92^ 2018	CPC/preintervention-postintervention	Rate/%	23.9%	20.4%	−3.5%^c^	Mean No. of pills		17	15	−2^c^
Anhalt et al,^93^ 2019	CPC/preintervention-postintervention	NA	NA	NA	NA	Mean MME		72.6	69.1	−3.5
Yang et al,^94^ 2020	CPC/preintervention-postintervention	Rate/%	42%	35.5%	−6.5%^a^	NA		NA	NA	NA
Delgado et al,^37^ 2018A	EMR_QC/ITS	NA	NA	NA	NA	6-MSC/No. of oxycodone		7.6	10.0	+2.4
Delgado et al,^37^ 2018B	EMR_QC/ITS	NA	NA	NA	NA	6-MSC/No. of oxycodone		12.3	11.6	−0.7
Slovis et al,^95^ 2018	EMR_QC/ITS	NA	NA	NA	NA	6-MSC/% >3 d		64.2%	14.1%	−50.1%^a^
Beauchamp et al,^96^ 2020	EMR_QC/ITS	NA	NA	NA	NA	6-MSC/MEDD		35.7	31.1	−4.6
Villwock et al,^97^ 2020	EMR_QC/ITS	NA	NA	NA	NA	6-MSC/MME		105.0	116.4	+11.4
Carlson et al,^98^ 2020	EMR_QC/ITS	NA	NA	NA	NA	6-MSC/No. of pills		20.6	17.8	−2.8^a^
Zwank et al,^99^ 2017	EMR_QC/preintervention-postintervention	No. of prescriptions	3331	3688	+357	Mean (SD) No. of pills		15.3 (5.3)	15.8 (7.3)	+0.5
Santistevan et al,^39^ 2018	EMR_QC/preintervention-postintervention	NA	NA	NA	NA	Median (IQR) No. of pills		20 (10-20)	15 (10-20)	−5^a^
Schwartz et al,^40^ 2019	EMR_QC/preintervention-postintervention	NA	NA	NA	NA	Mean (SD) oxycodone No. of pills		13.8 (5.1)	10.8 (5.6)	−3^a^
Smalley et al,^100^ 2020	EMR_QC/preintervention-postintervention	Rate/%	14.4%	7.4%	−7%^a^	% > 30 MEDD		4.3%	0.3%	−4%^a^
Johnson et al,^101^ 2020	EMR_QC/preintervention-postintervention	Rate/%	12.2%	11.5%	−0.7%^a^	Mean MEDD		31.7	28.8	−2.9^a^
Shelton et al,^102^ 2020	EMR_QC/preintervention-postintervention	NA	NA	NA	NA	% >155 MME pills		50%	31%	−19%^c^
Kim et al,^103^ 2019	PT/cohort study	Rate/%	42%	50%	+8%	Mean MME		101.7	102.2	+0.5
Pugh et al,^104^ 2020	PT/cohort study	Rate/%	21.7%	16.0%	−5.7%	NA		NA	NA	NA

Abbreviations: CPC, clinician peer comparison opioid prescribing; EMR_QC, electronic medical record quantity changes; EPG, educating, policy, or guidelines evaluation; ITS, interrupted time series; MEDD, morphine equivalent daily dosage in mg; MME, milligram morphine equivalent; 6-MSC, 6-month step change; NA, not applicable; PDMP, prescription drug monitoring program or state law; PT, physical therapy consultation; RCT, randomized clinical trial.

^a^ *P* < .05.

^b^ Interrupted time series analyzed for opioid prescription rates only.

^c^ Significance not reported in the study.

### Primary Meta-Analysis: Interventions to Reduce Rate and/or Prescribed Opioid Quantity

**Figure 2.**
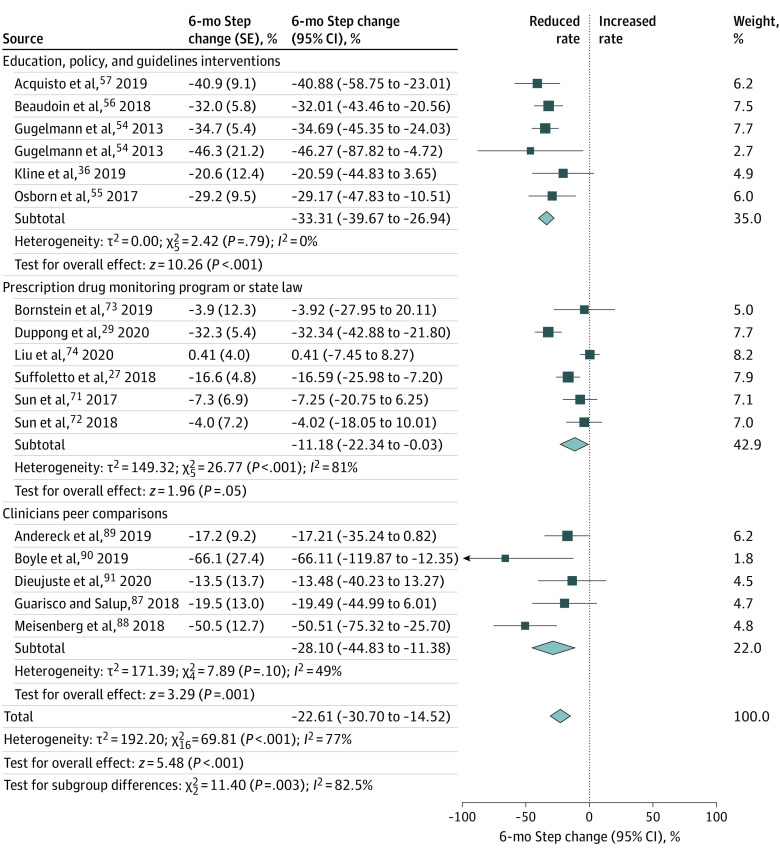
Forest Plots of the 6-Month Step Change for Opioid Prescription Rate in Interrupted Time Series Studies All analyses were conducted using an inverse variance weighting method and a random-effects model. Box widths are proportional to weights of individual studies in the meta-analysis. Diamond widths are 95% CIs of the summary estimates of the intervention outcome.

**Figure 3.**
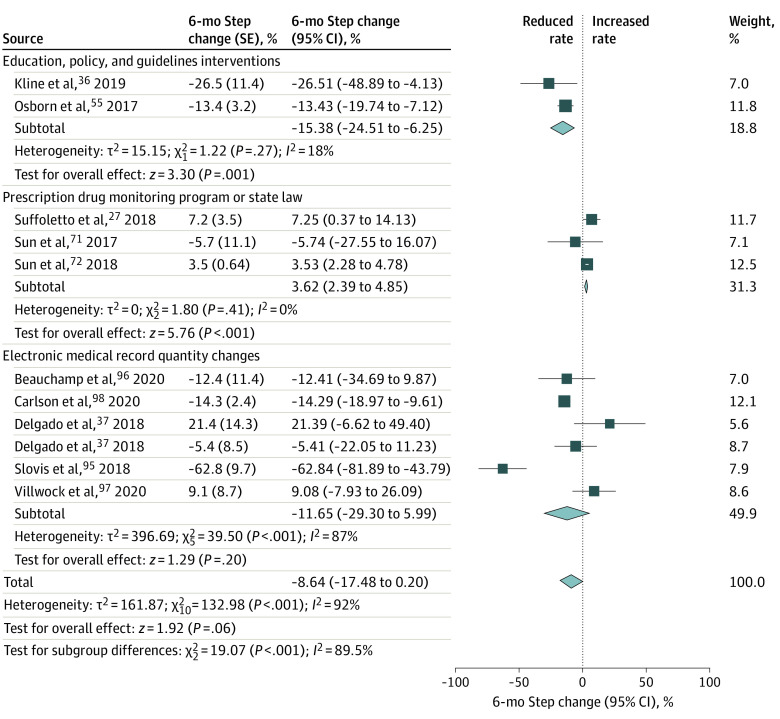
Forest Plots of the 6-Month Step Change for Opioid Prescription Quantity in Interrupted Time Series Studies All analyses were conducted using an inverse variance weighting method and a random-effects model. Box widths are proportional to weights of individual studies in the meta-analysis. Diamond widths are 95% CIs of the summary estimates of the intervention outcome.

Forty-five of 63 studies could be included in the meta-analysis for 1 or both components of our main outcome: 36 (80%) studies for the prescription rate^11,26,27,28,29,30,31,35,53,54,55,56,57,58,62,63,64,65,66,70,71,72,73,74,78,81,83,85,86,87,88,89,90,100,102,103^ and 23 (51%) for the prescribed opioid quantity^11,26,29,30,35,36,38,39,54,56,63,64,70,71,81,83,84,85,94,95,96,97,98^ at ED discharge. The interventions were associated with a significant reduction in the opioid prescription rate in both ITS (6-month step change, −22.61% [95% CI, −30.70% to −14.52%]; *I*^2^ = 77%) (Figure 2) and other study designs (OR, 0.56 [95% CI, 0.45 to 0.70]; *I*^2^ = 99%) (eFigure 4 in the Supplement). Interventions were not associated with a significant reduction in prescribed opioid quantity in ITS studies (6-month step change, −8.64% [95% CI, −17.48% to 0.20%]; *I*^2^ = 92%) (Figure 3). However, for other study designs, a small albeit significant reduction in prescribed quantity was found (SMD, −0.30 [95% CI, −0.51 to −0.09]; *I*^2^ = 100%) (eFigure 5 in the Supplement).

### Intervention Category Analysis

Not all intervention categories had data for each outcome. For ITS design, no EMR-QC and PT studies reported data on prescription rate, and no CPC and PT studies reported data on prescription quantities. For other study designs, only PT studies did not report data on prescription quantities.

There were significant differences in the reduction of the opioid prescription rates between intervention categories in ITS study designs (*P* = .003). The EPG intervention (6-month step change, −33.31% [95% CI, −39.67% to −26.94%]; *I*^2^ = 0%) provided a larger reduction rate (*P* < .001) compared with PDMP (6-month step change, −11.18% [95% CI, −22.34% to −0.03%]; *I*^2^ = 81%) (Figure 2). For the other study designs, EPG (OR, 0.47 [95% CI, 0.33-0.69]; *I*^2^ = 99%), CPC (OR, 0.46 [95% CI, 0.29-0.72]; *I*^2^ = 96%), and PDMP (OR, 0.61 [95% CI, 0.44-0.86]; *I*^2^ = 96%) showed a statistically significant reduction in the opioid prescription rate (*P* < .001) compared with PT (OR, 0.98 [95% CI, 0.49-1.95]; *I*^2^ = 75%) and EMR-QC (OR, 0.94 [95% CI, 0.88-0.99]; *I*^2^ = 99%), which did not show significant reduction (eFigure 4 in the Supplement).

In ITS studies, a statistically significant reduction in prescribed opioid quantities was demonstrated for EPG interventions (*P* < .001) (6-month step change, −15.38% [95% CI, −24.51% to −6.25%]; *I*^2^ = 18%) compared with PDMP (6-month step change, 3.62% [95% CI, 2.39% to 4.85%]; *I*^2^ = 0%) and EMR_QC (6-month step change, −11.65% [95% CI, −29.30% to 5.99%]; *I*^2^ = 87%) (Figure 3). In other study designs, PDMP (SMD, −0.37 [95% CI, −0.58 to −0.15]; *I*^2^ = 95%) showed a significant reduction in prescribed opioid quantities compared with EPG (SMD, −0.07 [95% CI, −0.15 to 0.02]; *I*^2^ = 33%), CPC (SMD, −0.51 [95% CI, −1.10 to 0.08]; *I*^2^ = 100%), and EMR_QC (SMD, −0.20 [95% CI, −0.47 to 0.07]; *I*^2^ = 100%) interventions (*P* = .03) (eFigure 5 in the Supplement).

### Secondary Outcomes

Patients’ level of pain relief was not reported in any study. Patients’ satisfaction level with their opioid prescriptions was presented in 4 studies: 1 had a very low survey response rate (1.9%),^57^ 2 reported no impact,^87,89^ and 1 found a slight gain (from 52% to 61%).^88^ One study^82^ reported on patients’ need for additional opioid prescriptions after the intervention and found no change for this outcome.

### Sensitivity Analysis

The 1-year step results of segmented ITS analysis for the opioid prescription rate and quantity are presented in eFigures 6 and 7 in the Supplement. The overall results of interventions and intervention categories were similar to those reported for the 6-month step change.

Removing the ITS studies with a high risk of bias (serious, critical, and high risk) left 10 studies^27,36,54,55,56,57,88,89,90,91^ with a moderate risk of bias and did not significantly alter our results (eFigures 8 and 9 in the Supplement). The RCT study (low risk of bias),^86^ with its CPC intervention, demonstrated a significant reduction in opioid prescription rate (−5.5%) and quantity (−8 milligram morphine equivalent).

### Publication Bias

Relative visual asymmetry was found in the 4 funnel plots for both outcomes and study designs (ITS vs others), suggesting a possible publication bias (eFigures 10-13 in the Supplement). However, Egger test results were nonsignificant for 6-month step change in the opioid prescription rate for ITS studies (Egger regression intercept: −1.65 [SE, 1.14]; *P* = .17), in the opioid prescription rate for RCT, preintervention-postintervention, and cohort studies (Egger regression intercept: 0.24 [SE, 3.4]; *P* = .94), in the prescribed opioid quantity for ITS studies (Egger regression intercept: −2.22 [SE, 1.22]; *P* = .10), and in the prescribed opioid quantity for RCT and preintervention-postintervention studies (Egger regression intercept: 7.08 [SE, 5.92]; *P* = .26).

## Discussion

This meta-analysis of recent studies originating mostly from the US (87%) showed that specific interventions were associated with a reduction of opioid prescription rates, but interventions in general were limited in reducing prescribed opioid quantities. In a subgroup analysis of the more robust ITS study designs, we showed that EPG interventions resulted in a larger prescription rate reduction compared with PDMP interventions. In addition, only EPG interventions were associated with a reduction in prescribed opioid quantities in ITS designs. Insufficient data were available on patient-centered secondary outcomes to reach any conclusion.

### Risk of Bias

We included 10 ITS studies that presented a moderate risk of bias and a single RCT study that exhibited a low risk of bias. For the remaining studies, the risk of bias was generally high, mainly because of the nature of their preintervention-postintervention designs. Interrupted time series studies are among the strongest evaluative designs when randomization is not possible; they are considered a robust design commonly used to evaluate the impact of interventions and programs implemented in health care settings.^105^ Given that they are frequently undertaken in real-world settings, ITS studies may have stronger external validity.^106^

We excluded studies conducted in populations other than patients discharged from the ED with pain or in pediatric populations because the intervention impact might be different from that obtained in adults discharged from the ED. We also excluded case reports and case series because of the high risk of bias associated with these designs. We found no significant publication bias, because all Egger test results were nonsignificant. Overall, significant heterogeneity was expected because of the diverse intervention categories and study designs. However, the heterogeneity was generally lower for intervention categories in ITS design studies, particularly for EPG interventions (*I*^2^ = 0% and *I*^2^ = 18%).

In a sensitivity analysis, we excluded studies at high risk of bias and found that exclusion did not significantly change our main results (significant reduction of the prescription rate and a trend for a reduction in prescribed opioid quantities), which demonstrates their consistency. This association also persisted in the 1-year sensitivity analysis. Moreover, most studies (90%) corroborated the association between interventions and prescription rate reductions. This finding suggests that our results are consistent for this outcome.

### Comparison With Previous Systematic Reviews

Systematic reviews of interventions to decrease opioid prescribing (rate or quantity) after surgery have demonstrated similar results.^107,108^ Although no PDMP or PT interventions were found in these reviews, they identified a type of intervention that relied exclusively on patient education. We have not encountered interventions of this nature in the present review.

Two systematic reviews^109,110^ specifically looked at US prescription drug monitoring programs in the postsurgical setting and found mixed results; they were not able to perform a meta-analysis of the reviewed studies. Similarly, other reviews not limited to the ED^111,112,113^ reported mixed results but also showed less effect in the ED and a lack of data on potential harms: no pain relief, increased ED revisits, and physicians changing their prescriptions to opioids not on the prescription drug monitoring programs (usually weaker). We included state laws as interventions in our PDMP category. Davis et al^114^ concluded that no data are available on the association between legislation and opioid-related morbidity or mortality and unintended negative outcomes. In our review, PDMP interventions demonstrated a small reduction of opioid prescription rate or quantity. However, in more robust ITS studies, PDMP interventions were associated with a small increase in the quantity of opioids prescribed. In addition, Moride et al^111^ found that prescription drug monitoring programs were mostly performed when abuse and diversions were suspected; these programs could thus reduce “drug-shopping” (obtaining multiple opioid prescriptions for the same problem). However, physicians being reassured that the patient is not seeking the drug might explain the increase in opioid quantities prescribed in PDMP studies. The ED’s rapid pace may prevent it from being an environment conducive to this intervention strategy.

Unexpectedly, the EMR_QC interventions that were specifically designed to reduce the quantity of opioids per prescription showed no significant reduction. Other reviews^107,115^ have reported a similar lack of an association between interventions and reduced prescriptions. However, this approach has been useful in postoperative settings^116^ and in ambulatory contexts when associated with state prescribing limits.^117^ The heterogeneity of the EMR_QC studies included in our review was high and may have contributed to the results; in some studies, the default EMR quantity of opioids to prescribe was reduced,^37,40,95,98^ whereas in others it was simply removed.^39,97,99^

The reduction observed for EPG interventions was somewhat unexpected considering that another review^118^ demonstrated that physicians’ adherence to guidelines was low for chronic noncancer pain treated with opioids. However, in that review, none of the included studies was mainly conducted in an ED setting, and EPG interventions led in an institutional context were found to be associated with a reduction in opioid prescriptions. Interestingly, some of these studies^115,119^ also included interventions focused on patients and other clinicians. Other reviews^115,120^ proposed that a multimodal educational strategy simultaneously targeting clinicians, patients, and other collaborators was the most promising approach to improve appropriate opioid use. However, they also showed numerous negative outcomes associated with strategies aiming to improve opioid use such as patients not receiving opioid prescriptions, overdose increase, naloxone-associated stigma, shifting the opioid crisis to a neighboring region, changing to another opioid class, and even increases in dose and proportion of prescribed opioids. These reviews also concluded that it would be ideal “to develop a method for reliably predicting the amount of opioid (if any) a patient may consider adequate for pain relief.”^119^^(p70)^

It is noteworthy to observe that there were almost no data on important patient-centered outcomes such as pain control level, satisfaction level, and additional opioid prescription needs across the 63 studies included in our systematic review. This finding can be explained primarily by the retrospective design of most of the studies. Considering the potential negative effect of these interventions on patient-centered outcomes, this outcome is a major knowledge gap. In the future, it is imperative to evaluate interventions designed to reduce opioid prescribing in a prospective manner and to integrate patient-centered outcomes.

### Limitations

Our review is limited by the quality of the included studies. However, our findings were consistent in the sensitivity analysis in which studies with a high risk of bias were excluded. Furthermore, 90% of included studies reported a reduction of prescription rate from almost all intervention categories. The intervention categories varied enormously on several parameters such as the type of intervention included, the way in which the intervention was implemented, the study design used, the type of outcomes measured, and the duration of the follow-up. Therefore, caution is warranted regarding the generalization of these categories. The heterogeneity (*I*^2^ value) was low for several categories in ITS studies except for PDMP studies on the rate of opioid prescribing. However, it was high for studies involving other study designs, suggesting high heterogeneity within preintervention-postintervention study designs. Some conclusions are limited by the small number of studies in the subgroup analyses. There were no ITS studies using EMR_QC for opioid prescription rate or using CPC for opioid prescribed quantity and no PT studies for either outcome. This lack of data limits our conclusions for these intervention categories. Furthermore, most studies were performed in the US and may not be generalizable to other health care systems. Physical therapy was the only type of intervention study in which participants were allocated at the physician discretion, leading to possible allocation bias. Except for ITS studies that account for secular tendency, the findings of most studies reflected the trend of decreased opioid prescribing in the ED within the past 10 years.^6,7,8^

## Conclusions

The findings of this meta-analysis suggest that specific interventions may be better at reducing the rate (to a lesser extent in reducing the quantity) of prescribed opioids to patients discharged from the ED. Therefore, policy makers and clinicians should probably focus their efforts on these more promising approaches to reduce prescribing rates. However, researchers should address the important knowledge gap on the global effect of these interventions on patient-centered outcomes and use novel approaches to reduce the opioid quantity per prescription to patients discharged from the ED.

## Notes

Supplement. **eMethods 1.** Detailed Search Strategy and Search Term Alternatives for Each Database **eMethods 2.** Description of the Interventions Design to Reduce Opioid Prescription and How They Were Categorized **eFigure 1.** Example of the 6-mo Step Changes Representation From Interrupted Time Series Data **eFigure 2.** Risk of Bias Assessed With the ROBINS-I Tool for ITS and Preintervention-Postintervention Study Designs **eFigure 3.** Risk of Bias Assessed With the EPOC Risk of Bias Tool for the RCT and Cohort Study Designs **eFigure 4.** Forest Plots of Opioid Prescription Rate in RCT, Preintervention-Postintervention, and Cohort Studies **eFigure 5.** Forest Plots of Opioid Prescription Quantity in RCT, Preintervention-Postintervention, and Cohort Studies **eFigure 6.** Forest Plot of the 1-y Step Change in Opioid Prescription Rate for ITS Studies **eFigure 7.** Forest Plot of the 1-y Step Change in Prescribed Opioid Quantity for ITS Studies **eFigure 8.** Forest Plot of the 6-mo Step Change in Opioid Prescription Rate for ITS Studies Excluding Studies at High Risk of Bias **eFigure 9.** Forest Plot of the 6-mo Step Change in Prescribed Opioid Quantity for ITS Studies Excluding Studies at High Risk of Bias **eFigure 10.** Funnel Plot With Pseudo 95% CIs of the 6-mo Step Change in the Opioid Prescription Rate for ITS Studies **eFigure 11.** Funnel Plot With Pseudo 95% CIs of the 6-mo Step Change in the Opioid Prescription Rate for RCT, Preintervention-Postintervention, and Cohort Studies **eFigure 12.** Funnel Plot With Pseudo 95% CIs of the 6-mo Step Change in the Prescribed Opioid Quantity for ITS Studies **eFigure 13.** Funnel Plot With Pseudo 95% CIs of the 6-mo Step Change in the Prescribed Opioid Quantity for RCT and Preintervention-Postintervention Studies
eReferences
