# Supplementary material for: Evaluation of Interventions to Reduce Opioid Prescribing for Patients Discharged From the Emergency Department: A Systematic Review and Meta-analysis
Source: JAMA Netw Open. 2022 Jan 13;5(1):e2143425. doi: 10.1001/jamanetworkopen.2021.43425 (PMC8759006; doi:10.1001/jamanetworkopen.2021.43425)
Supplement: Supplement. — eMethods 1. Detailed Search Strategy and Search Term Alternatives for Each Database eMethods 2. Description of the Interventions Design to Reduce Opioid Prescription and How They Were Categorized eFigure 1. Example of the 6-mo Step Changes Representation From Interrupted Time Series Data eFigure 2. Risk of Bias Assessed With the ROBINS-I Tool for ITS and Preintervention-Postintervention Study Designs eFigure 3. Risk of Bias Assessed With the EPOC Risk of Bias Tool for the RCT and Cohort Study Designs eFigure 4. Forest Plots of Opioid Prescription Rate in RCT, Preintervention-Postintervention, and Cohort Studies eFigure 5. Forest Plots of Opioid Prescription Quantity in RCT, Preintervention-Postintervention, and Cohort Studies eFigure 6. Forest Plot of the 1-y Step Change in Opioid Prescription Rate for ITS Studies eFigure 7. Forest Plot of the 1-y Step Change in Prescribed Opioid Quantity for ITS Studies eFigure 8. Forest Plot of the 6-mo Step Change in Opioid Prescription Rate for ITS Studies Excluding Studies at High Risk of Bias eFigure 9. Forest Plot of the 6-mo Step Change in Prescribed Opioid Quantity for ITS Studies Excluding Studies at High Risk of Bias eFigure 10. Funnel Plot With Pseudo 95% CIs of the 6-mo Step Change in the Opioid Prescription Rate for ITS Studies eFigure 11. Funnel Plot With Pseudo 95% CIs of the 6-mo Step Change in the Opioid Prescription Rate for RCT, Preintervention-Postintervention, and Cohort Studies eFigure 12. Funnel Plot With Pseudo 95% CIs of the 6-mo Step Change in the Prescribed Opioid Quantity for ITS Studies eFigure 13. Funnel Plot With Pseudo 95% CIs of the 6-mo Step Change in the Prescribed Opioid Quantity for RCT and Preintervention-Postintervention Studies eReferences [file jamanetwopen-e2143425-s001.pdf]

Supplementary Online Content

Daoust R, Paquet J, Marquis M, et al. Evaluation of interventions to reduce opioid prescribing for patients discharged from the emergency department: a systematic review and meta-analysis. *JAMA Netw Open*. 2022;5(1):e2143425. doi:10.1001/jamanetworkopen.2021.43425

eMethods 1. Detailed Search Strategy and Search Term Alternatives for Each Database

eMethods 2. Description of the Interventions Design to Reduce Opioid Prescription and How They Were Categorized

eFigure 1. Example of the 6-mo Step Changes Representation From Interrupted Time Series Data

eFigure 2. Risk of Bias Assessed With the ROBINS-I Tool for ITS and Preintervention-Postintervention Study Designs

eFigure 3. Risk of Bias Assessed With the EPOC Risk of Bias Tool for the RCT and Cohort Study Designs

eFigure 4. Forest Plots of Opioid Prescription Rate in RCT, Preintervention-Postintervention, and Cohort Studies

eFigure 5. Forest Plots of Opioid Prescription Quantity in RCT, Preintervention-Postintervention, and Cohort Studies

eFigure 6. Forest Plot of the 1-y Step Change in Opioid Prescription Rate for ITS Studies

eFigure 7. Forest Plot of the 1-y Step Change in Prescribed Opioid Quantity for ITS Studies

eFigure 8. Forest Plot of the 6-mo Step Change in Opioid Prescription Rate for ITS Studies Excluding Studies at High Risk of Bias

eFigure 9. Forest Plot of the 6-mo Step Change in Prescribed Opioid Quantity for ITS Studies Excluding Studies at High Risk of Bias

eFigure 10. Funnel Plot With Pseudo 95% CIs of the 6-mo Step Change in the Opioid Prescription Rate for ITS Studies

eFigure 11. Funnel Plot With Pseudo 95% CIs of the 6-mo Step Change in the Opioid Prescription Rate for RCT, Preintervention-Postintervention, and Cohort Studies

eFigure 12. Funnel Plot With Pseudo 95% CIs of the 6-mo Step Change in the Prescribed Opioid Quantity for ITS Studies

eFigure 13. Funnel Plot With Pseudo 95% CIs of the 6-mo Step Change in the Prescribed Opioid Quantity for RCT and Preintervention-Postintervention Studies

eReferences

This supplementary material has been provided by the authors to give readers additional information about their work.

eMethods 1. Detailed Search Strategy and Search Term Alternatives for Each Database

Search Strategy

<u>Ovid MEDLINE</u>

1. exp Narcotics/	
2. exp Opiate Alkaloids/	
3. opioid*.mp.	
4. opiate*.mp.	
5. opium*.mp.	
6. narcotic*.mp.	
7. alfentanil.mp.	
8. buprenorphine.mp.	
9. butorphanol.mp.	
10. codeine.mp.	
11. dextropropoxyphene.mp.	
12. fentan?l.mp.	
13. hydrocodone.mp.	
14. hydromorphone.mp.	
15. levorphanol.mp.	
16. meperidine.mp.	
17. methadone.mp.	
18. morphine.mp.	
19. nalbuphine.mp.	
20. normethadone.mp.	
21. oxycodone.mp.	
22. pentazocine.mp.	
23. pethidine.mp.	
24. propoxyphene.mp.	
25. remifentanil.mp.	
26. sufentanil.mp.	
27. tapentadol.mp.	
28. tramadol.mp.	
29. or/1-28	
30. emerg*.tw,kf.	
31. emergency medical services/ or emergency service, hospital/ or emergency services, psychiatric/	
32. or/30-31	
33. Drug Therapy/	
34. Drug Dosage Calculations/	
35. exp Drug Prescriptions/	
36. Inappropriate Prescribing/	
37. Practice Patterns, Physicians'/	

© 2022 Daoust R et al. *JAMA Network Open*.

38. prescription*.tw,kf.	
39. prescrib*.tw,kf.	
40. ((drug* or medication*) adj4 (discard* or dispos*)).tw,kf.	
41. or/33-40	
42. 29 and 32 and 41	

EMBASE

1. exp narcotic analgesic agent/	
2. opioid*.mp.	
3. opiate*.mp.	
4. opium*.mp.	
5. narcotic*.mp.	
6. alfentanil.mp.	
7. buprenorphine.mp.	
8. butorphanol.mp.	
9. codeine.mp.	
10. dextropropoxyphene.mp.	
11. fentan?l.mp.	
12. hydrocodone.mp.	
13. hydromorphone.mp.	
14. levorphanol.mp.	
15. meperidine.mp.	
16. methadone.mp.	
17. morphine.mp.	
18. nalbuphine.mp.	
19. normethadone.mp.	
20. oxycodone.mp.	
21. pentazocine.mp.	
22. pethidine.mp.	
23. propoxyphene.mp.	
24. remifentanil.mp.	
25. sufentanil.mp.	
26. tapentadol.mp.	
27. tramadol.mp.	
28. or/1-27	
29. emerg*.tw,kw.	
30. emergency health service/	
31. hospital emergency service/	
32. psychiatric emergency service/	
33. or/29-32	
34. dose calculation/	
35. drug misuse/	
36. prescription/	
37. exp inappropriate prescribing/	

© 2022 Daoust R et al. *JAMA Network Open*.

38. clinical practice/	
39. drug utilization/	
40. prescription*.tw,kw.	
41. prescrib*.tw,kw.	
42. ((drug* or medication*) adj4 (discard* or dispos*)).tw,kw.	
43. (therap* or treatment*).ti,kw.	
44. or/34-43	
45. 28 and 33 and 44	

CENTRAL

(ebmz|All EBM Reviews - Cochrane DSR, ACP Journal Club, DARE, CCA, CCTR, CMR, HTA, and NHSEED)

1. exp Narcotics/	
2. exp Opiate Alkaloids/	
3. opioid*.tw.	
4. opiate*.tw.	
5. opium*.tw.	
6. narcotic*.tw.	
7. alfentanil.tw.	
8. buprenorphine.tw.	
9. butorphanol.tw.	
10. codeine.tw.	
11. dextropropoxyphene.tw.	
12. fentan?l.tw.	
13. hydrocodone.tw.	
14. hydromorphone.tw.	
15. levorphanol.tw.	
16. meperidine.tw.	
17. methadone.tw.	
18. morphine.tw.	
19. nalbuphine.tw.	
20. normethadone.tw.	
21. oxycodone.tw.	
22. pentazocine.tw.	
23. pethidine.tw.	
24. propoxyphene.tw.	
25. remifentanil.tw.	
26. sufentanil.tw.	
27. tapentadol.tw.	
28. tramadol.tw.	
29. or/1-28	
30. emerg*.tw,kw.	

31. Drug Therapy/	
32. Drug Dosage Calculations/	
33. exp Prescription Drug Misuse/	
34. exp Drug Prescriptions/	
35. Inappropriate Prescribing/	
36. Practice Patterns, Physicians'/	
37. prescription*.ti,ab,hw.	
38. prescrib*.ti,ab,hw.	
39. ((drug* or medication*) adj4 (discard* or dispos*)).ti,ab,hw.	
40. or/31-39	
41. 29 and 30 and 40	

Psycinfo

Any Field: analgesic drugs OR
Any Field: narcotic drugs OR
Any Field: opioid* OR
Any Field: narcotic* OR
Any Field: opium* OR
Any Field: opiates OR
Any Field: morphin* OR
Any Field: codeine* OR
Any Field: oxycodo* OR
Any Field: hydroco* OR
Any Field: hydromor* OR
Any Field: fentan* OR
Any Field: meperidin* OR
Any Field: tramadol* OR
Any Field: pethidine* OR
Any Field: methadone* OR
Any Field: alfentanil* OR
Any Field: buprenorphine* OR
Any Field: butorphanol* OR
Any Field: dextropropoxyphene* OR
Any Field: levorphanol* OR
Any Field: nalbuphine* OR
Any Field: normethadone* OR
Any Field: pentazocine* OR
Any Field: propoxyphene* OR
Any Field: remifentanil* OR
Any Field: sufentanil* OR
Any Field: tapentadol*

AND
Any Field: drug therapy OR

Any Field: drug dosages OR
Any Field: prescription drugs OR
Any Field: prescri*

AND
Any Field: emerg*

<u>Cinahl</u>

(MH “Narcotics+”) OR
(MH “Analgesics, Opioid+”) OR
(MH “Narcotic Antagonists+”) OR
TX opioid* OR
TX opiate* OR
TX opium* OR
TX narcotic* OR
TX alfentanil OR
TX buprenorphine OR
TX butorphanol OR
TX codeine OR
TX dextropropoxyphene OR
TX fentan?l OR
TX hydrocodone OR
TX hydromorphone OR
TX levorphanol OR TX meperidine OR
TX methadone OR
TX morphine OR
TX nalbuphine OR
TX normethadone OR
TX oxycodone OR
TX pentazocine OR
TX pethidine OR
TX propoxyphene OR
TX remifentanil OR
TX sufentanil OR
TX tapentadol OR
TX tramadol
AND

(MH “Drug Therapy”) OR
(MH “Prescriptions, Drug”) OR
(MH “Inappropriate Prescribing”) OR
(MH “Prescribing Patterns”) OR

© 2022 Daoust R et al. *JAMA Network Open*.

TI prescription* OR
AU prescription* OR
TI prescrib* OR
AB prescrib*
AND

TX emerg*

ClinicalTrials.gov

Prescription and Opioid and Emergency

Google Scholar

(opioid OR narcotic) AND (prescribing OR prescription) AND (emergency)

© 2022 Daoust R et al. *JAMA Network Open*.

eMethods 2. Description of the Interventions Design to Reduce Opioid Prescription and How They Were Categorized

Interventions	Detailed description	Category	Rational for categorization
Education	Grand rounds, journal clubs, lectures, conferences, meetings, one-on-one sessions, online modules, courses to opioid prescribers	EPG	Set of information given to change physicians' behavior that are not mandatory
Policy, Guidelines	Documents with guiding decision aims and criteria regarding opioid prescriptions for physicians	EPG	
Clinician Peer Comparison	Comparison/sharing of opioid prescription patterns with other physicians	CPC	Change in physicians' behavior that is influenced by his peers
Prescription Drug Monitoring Program	State prescription drug monitoring program within an electronic database used by physicians to adjust opioid prescribing that keeps track of controlled substance prescriptions	PDMP	Change in physicians' behavior that are mandatory
State Law	State law that specifically modified opioid prescription such as rescheduling of hydrocodone combination products from schedule III to schedule II	PDMP	
Electronic Medical Record Quantity Changes	Modifications of the electronic medical record system by the local healthcare organization to reduce or remove the default quantity of opioids to prescribe	EMR_QC	Change in healthcare organization to modify physicians' opioid prescribing
Physical Therapy	Other approach not directly aimed at physician opioid prescribing	PT	Physicians are not the main target of intervention

© 2022 Daoust R et al. *JAMA Network Open*.

eFigure 1. Example of the 6-mo Step Changes Representation From Interrupted Time Series Data

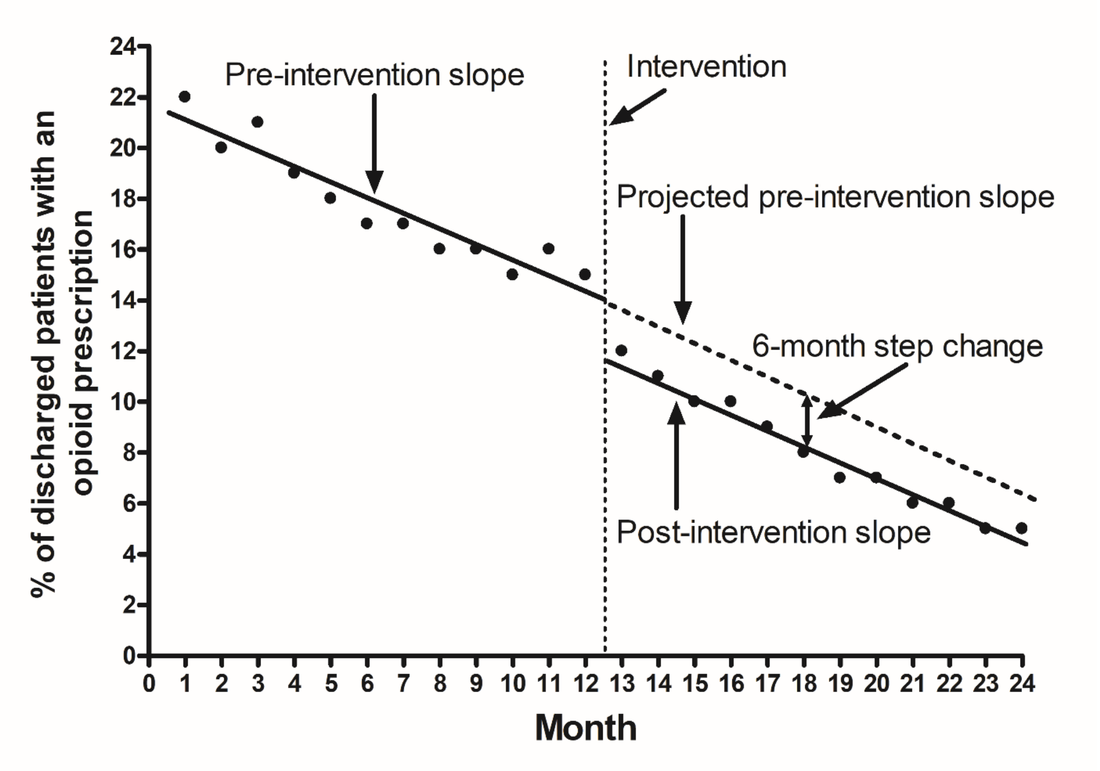

<u>ITS standardizing approach</u>

For time series data that were presented only in graphs, two independent raters (JP and MM) used WebPlotDigitizer (https://automeris.io/WebPlotDigitizer/) to extract data from plots. Intraclass correlation coefficients (ICC) were used to assess interrater reliability. Average data extracted from the graphs were used for ITS analysis when interrater reliability was excellent (ICC>90).[1]

To produce an intervention effect that integrated both the step (immediate) and slope (sustained) changes in ITS studies (if at least three data points before and three data points after the intervention were available), we used segmented time-series regression analyses while taking into account secular time trends and autocorrelation[2] to calculate the 6-month step change.[3] It is calculated as the difference between the level 6-month after intervention on the post-intervention fitted slope and the level predicted by the pre-intervention time trend (eFigure 1 in the supplement). The Durbin-Watson test was used before the segmented time series to assess autocorrelation and, if positive, was added to the model. Since follow-up times varied in ITS studies and the optimal follow-up time is not known, a consensus was obtained across 6 reviewers (RD, JP, MM, JMC, DW, AC) that the 6-month step change would represent a good estimate of the combined effect of immediate and sustained change due to the intervention. For those studies that had fewer than 6 months of post-intervention follow-up, we selected the last available follow-up time point.

Since included ITS studies were not all on the same scale (some used proportion of patients with an opioid prescription and others used crude number of patients), the effect sizes could not be compared directly. Therefore, we calculated a relative 6-month step change by dividing the coefficients and the standard errors by the average of baseline data points and multiplying by 100.[2]

© 2022 Daoust R et al. *JAMA Network Open*.

eFigure 2. Risk of Bias Assessed With the ROBINS-I Tool for ITS and Preintervention-Postintervention Study Designs

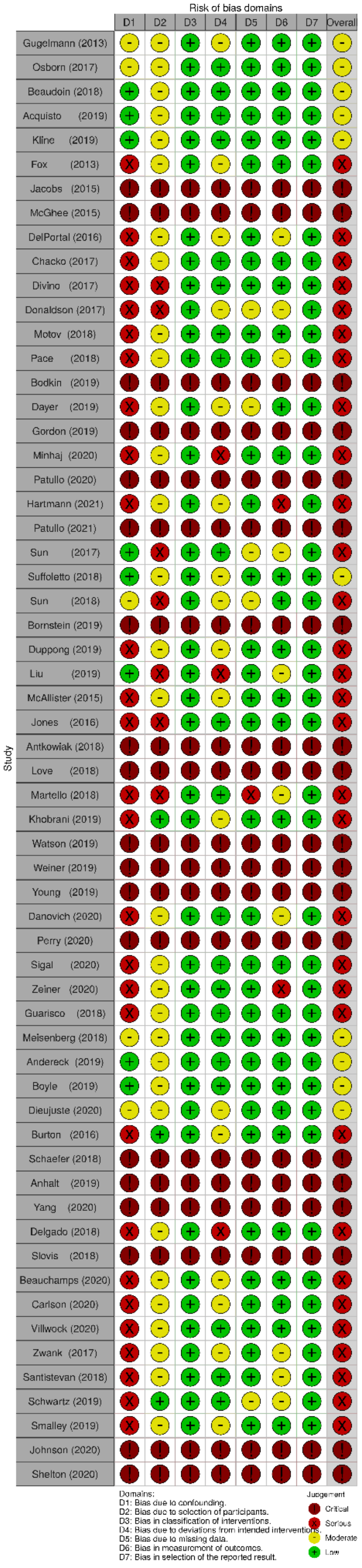

© 2022 Daoust R et al. *JAMA Network Open*.

eFigure 3. Risk of Bias Assessed With the EPOC Risk of Bias Tool for the RCT and Cohort Study Designs

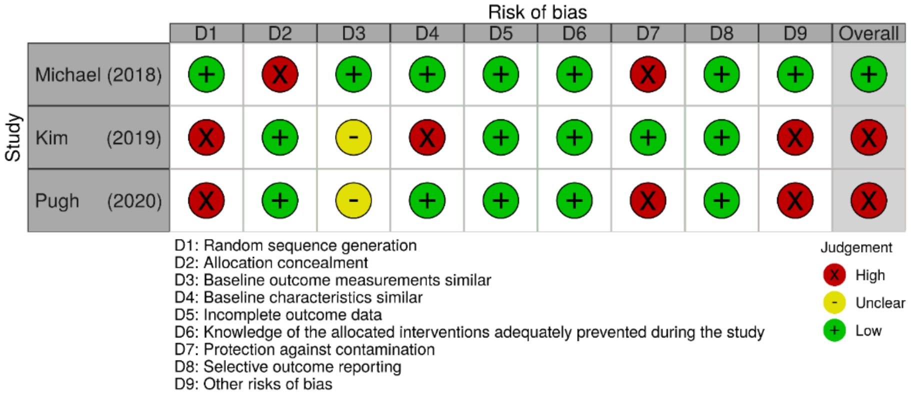

© 2022 Daoust R et al. *JAMA Network Open*.

eFigure 4. Forest Plots of Opioid Prescription Rate in RCT, Preintervention-Postintervention, and Cohort Studies

Study or Subgroup	Post-Intervention Events	Post-Intervention Total	Pre-Intervention Events	Pre-Intervention Total	Weight	Odds Ratio IV, Random, 95% CI
**3.1.1 Education, Policy, Guidelines interventions**						
Bodkin et al,(64) 2019	70	13029	279	13326	5.1%	0.25 [0.19, 0.33]
Dayer et al,(30) 2019	4263	27442	4389	26603	5.5%	0.93 [0.89, 0.97]
del Portal et al,(31) 2016	1229	4122	2392	4540	5.4%	0.38 [0.35, 0.42]
Fox et al,(57) 2013	65	153	302	515	4.8%	0.52 [0.36, 0.75]
Gordon et al,(65) 2019	352	7334	549	7280	5.4%	0.62 [0.54, 0.71]
Jacobs et al,(58) 2015	159	870	340	828	5.2%	0.32 [0.26, 0.40]
Minhaj et al,(66) 2020	173	284	212	307	4.9%	0.70 [0.50, 0.98]
Motov et al,(62) 2018	634	1471	1402	2044	5.4%	0.35 [0.30, 0.40]
Pace et al,(63) 2018	57	243	87	238	4.7%	0.53 [0.36, 0.79]
**Subtotal (95% CI)**		**54948**		**55681**	**46.3%**	**0.47 [0.33, 0.69]**
Total events	7002		9952			
Heterogeneity: Tau² = 0.31; Chi² = 536.69, df = 8 (P < 0.00001); I² = 99%						
Test for overall effect: Z = 3.91 (P < 0.0001)						
**3.1.2 Prescription Drug Monitoring Program or State Law**						
Danovoch et al,(27) 2020	642	22405	1366	22221	5.4%	0.45 [0.41, 0.50]
Khobrani et al,(29) 2019	64	134	62	134	4.4%	1.06 [0.66, 1.72]
Martello et al,(78) 2018	143	887	287	1335	5.2%	0.70 [0.56, 0.88]
McAllister et al,(74) 2015	84	356	69	354	4.8%	1.28 [0.89, 1.83]
Sigal et al,(83) 2021	2203	29848	5665	29255	5.5%	0.33 [0.31, 0.35]
Young et al,(81) 2019	9	58	51	164	3.3%	0.41 [0.19, 0.89]
**Subtotal (95% CI)**		**53688**		**53463**	**28.6%**	**0.61 [0.44, 0.86]**
Total events	3145		7500			
Heterogeneity: Tau² = 0.14; Chi² = 128.63, df = 5 (P < 0.00001); I² = 96%						
Test for overall effect: Z = 2.84 (P = 0.004)						
**3.1.3 Clinicians Peer Comparison**						
Burton et al,(11) 2016	2666	32118	16448	82241	5.5%	0.36 [0.35, 0.38]
Michael et al,(85) 2018	935	10724	4007	28224	5.5%	0.58 [0.54, 0.62]
**Subtotal (95% CI)**		**42842**		**110465**	**10.9%**	**0.46 [0.29, 0.72]**
Total events	3601		20455			
Heterogeneity: Tau² = 0.11; Chi² = 111.70, df = 1 (P < 0.00001); I² = 99%						
Test for overall effect: Z = 3.36 (P = 0.0008)						
**3.1.4 Physical Therapy consultation**						
Kim et al,(102) 2019	37	74	163	390	4.3%	1.39 [0.85, 2.29]
Pugh et al,(103) 2020	61	381	31	143	4.4%	0.69 [0.42, 1.12]
**Subtotal (95% CI)**		**455**		**533**	**8.7%**	**0.98 [0.49, 1.95]**
Total events	98		194			
Heterogeneity: Tau² = 0.19; Chi² = 3.96, df = 1 (P = 0.05); I² = 75%						
Test for overall effect: Z = 0.07 (P = 0.95)						
**3.1.5 Electronic Medical Record Quantity Changes**						
Johnson et al,(100) 2020	2432	21149	2646	21687	5.5%	0.94 [0.88, 0.99]
**Subtotal (95% CI)**		**21149**		**21687**	**5.5%**	**0.94 [0.88, 0.99]**
Total events	2432		2646			
Heterogeneity: Not applicable						
Test for overall effect: Z = 2.25 (P = 0.02)						
**Total (95% CI)**		**173082**		**241829**	**100.0%**	**0.56 [0.45, 0.70]**
Total events	16278		40747			
Heterogeneity: Tau² = 0.23; Chi² = 1760.22, df = 19 (P < 0.00001); I² = 99%						
Test for overall effect: Z = 5.08 (P < 0.00001)						
Test for subgroup differences: Chi² = 26.43, df = 4 (P < 0.0001), I² = 84.9%						

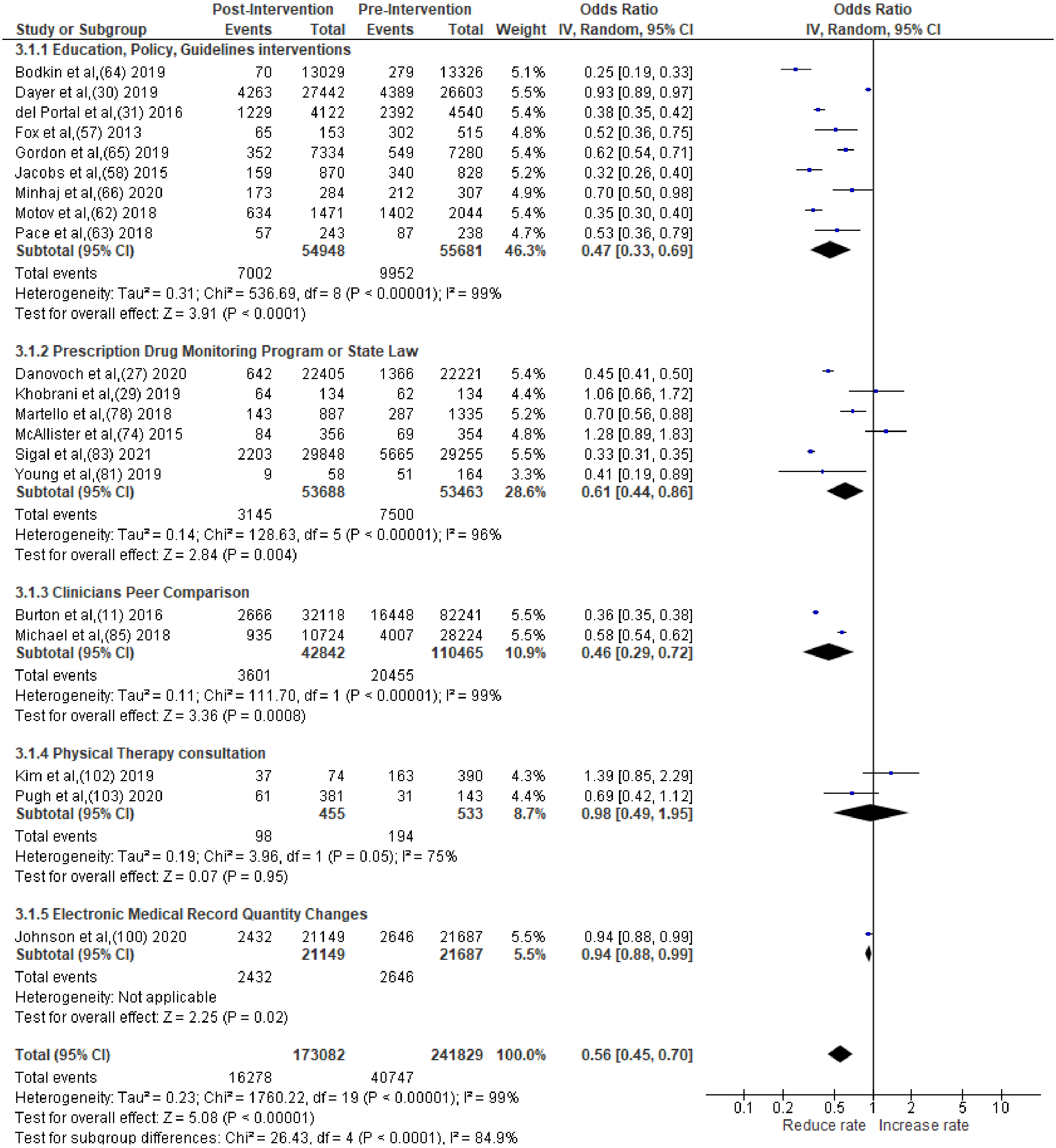

© 2022 Daoust R et al. *JAMA Network Open*.

eFigure 5. Forest Plots of Opioid Prescription Quantity in RCT, Preintervention-Postintervention, and Cohort Studies

Study or Subgroup	Post-Intervention Mean	Post-Intervention SD	Post-Intervention Total	Pre-Intervention Mean	Pre-Intervention SD	Pre-Intervention Total	Weight	Std. Mean Difference IV, Random, 95% CI
**4.1.1 Education, Policy, Guidelines interventions**								
Acquisto et al,(56) 2019	111.7	83.2	336	113.9	82.6	780	8.2%	-0.03 [-0.15, 0.10]
Bodkin et al,(64) 2019	31.8	15.6	70	37.7	26.6	279	7.5%	-0.24 [-0.50, 0.03]
Dayer et al,(30) 2019	28.4	18.7	4457	29.1	20.4	4601	8.4%	-0.04 [-0.08, 0.01]
Pace et al,(63) 2018	105.8	76.7	57	132.3	95.6	87	6.9%	-0.30 [-0.63, 0.04]
**Subtotal (95% CI)**			**4920**			**5747**	**31.0%**	**-0.07 [-0.15, 0.02]**
Heterogeneity: Tau² = 0.00; Chi² = 4.49, df = 3 (P = 0.21); I² = 33%								
Test for overall effect: Z = 1.55 (P = 0.12)								
**4.1.2 Prescription Drug Monitoring Program or State Law**								
Khobrani et al,(29) 2019	75	22.75	64	85	42.54	62	6.8%	-0.29 [-0.64, 0.06]
Sigal et al,(83) 2021	86.8	110.4	2203	104.9	72.7	5665	8.4%	-0.21 [-0.26, -0.16]
Young et al,(81) 2019	92.7	59.5	9	225.3	171.6	51	4.2%	-0.81 [-1.54, -0.09]
Zeiner et al,(84) 2020	83.3	29.7	4509	98.3	33.4	5798	8.4%	-0.47 [-0.51, -0.43]
**Subtotal (95% CI)**			**6785**			**11576**	**27.9%**	**-0.37 [-0.58, -0.15]**
Heterogeneity: Tau² = 0.03; Chi² = 65.79, df = 3 (P < 0.00001); I² = 95%								
Test for overall effect: Z = 3.36 (P = 0.0008)								
**4.1.3 Clinicians Peer Comparison**								
Burton et al,(11) 2016	13.5	3.3	26666	16.7	4.8	16448	8.4%	-0.81 [-0.83, -0.79]
Michael et al,(85) 2018	90	40	7440	97.5	33.4	19654	8.4%	-0.21 [-0.24, -0.19]
**Subtotal (95% CI)**			**34106**			**36102**	**16.9%**	**-0.51 [-1.10, 0.08]**
Heterogeneity: Tau² = 0.18; Chi² = 1232.10, df = 1 (P < 0.00001); I² = 100%								
Test for overall effect: Z = 1.71 (P = 0.09)								
**4.1.4 Electronic Medical Record Quantity Changes**								
Santistevan et al,(38) 2018	15	7.4	2400	16.7	7.4	4104	8.4%	-0.23 [-0.28, -0.18]
Schwartz et al,(39) 2019	10.8	5.6	106	13.8	5.1	102	7.4%	-0.56 [-0.83, -0.28]
Zwank et al,(98) 2017	15.8	7.3	3688	15.3	5.3	3331	8.4%	0.08 [0.03, 0.12]
**Subtotal (95% CI)**			**6194**			**7537**	**24.2%**	**-0.20 [-0.47, 0.07]**
Heterogeneity: Tau² = 0.05; Chi² = 88.45, df = 2 (P < 0.00001); I² = 98%								
Test for overall effect: Z = 1.48 (P = 0.14)								
**Total (95% CI)**			**52005**			**60962**	**100.0%**	**-0.30 [-0.51, -0.09]**
Heterogeneity: Tau² = 0.14; Chi² = 2618.01, df = 12 (P < 0.00001); I² = 100%								
Test for overall effect: Z = 2.77 (P = 0.006)								
Test for subgroup differences: Chi² = 8.85, df = 3 (P = 0.03), I² = 66.1%								

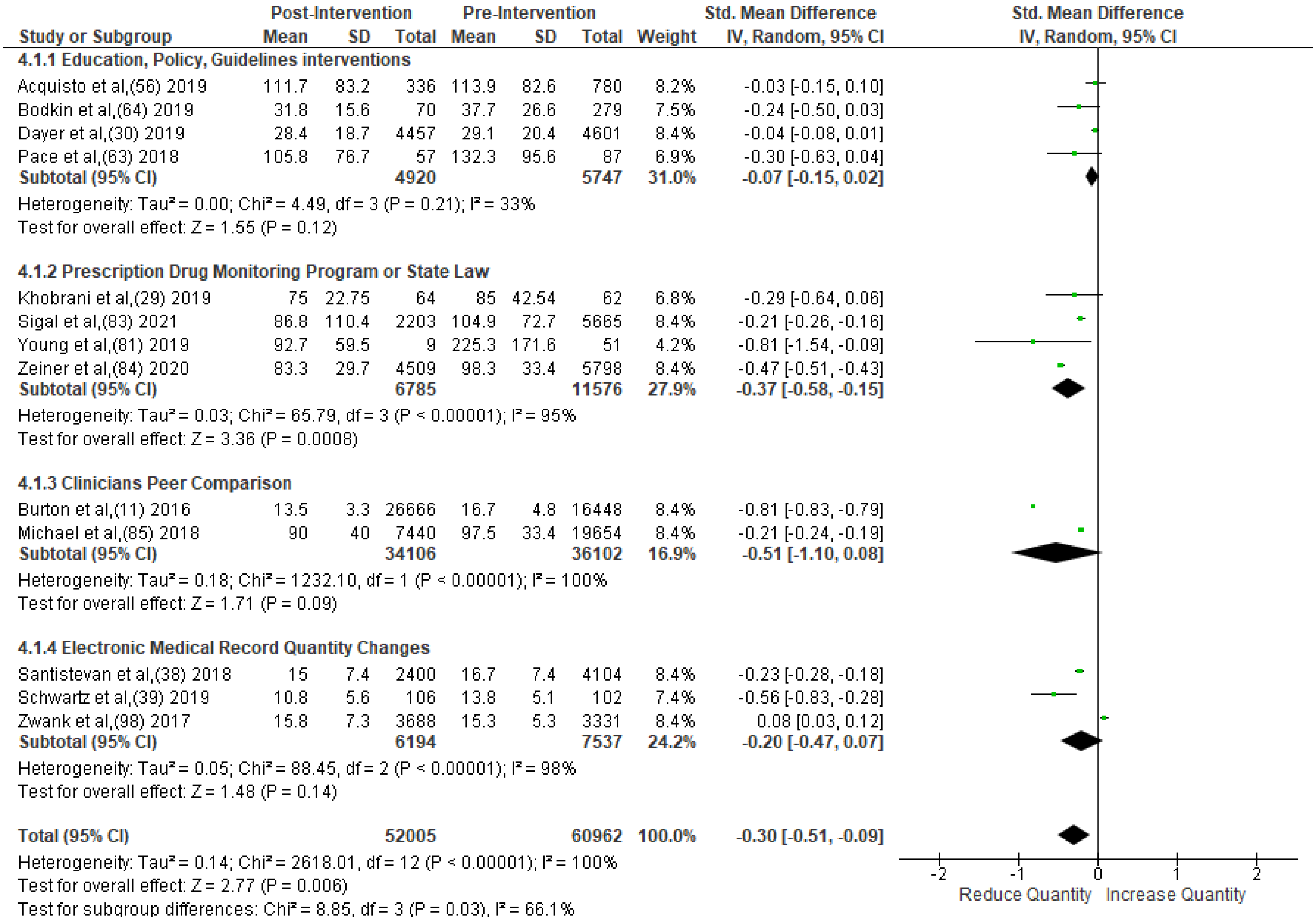

© 2022 Daoust R et al. *JAMA Network Open*.

eFigure 6. Forest Plot of the 1-y Step Change in Opioid Prescription Rate for ITS Studies

Study or Subgroup	1-year step change (%)	SE	Weight	1-year step change (%) IV, Random, 95% CI
**1.1.1 Education, Policy, Guidelines Interventions**				
Acquisto et al,(56) 2019	-57	15.5	5.3%	-57.00 [-87.38, -26.62]
Beaudoin et al,(55) 2018	-35.1	8.8	7.5%	-35.10 [-52.35, -17.85]
Gugelmann et al,(53) 2013a	-37.4	6.6	8.2%	-37.40 [-50.34, -24.46]
Gugelmann et al,(53) 2013b	-75.5	35.3	1.9%	-75.50 [-144.69, -6.31]
Kline et al,(35) 2019	-20.6	12.4	6.3%	-20.60 [-44.90, 3.70]
Osborn et al,(54) 2017	-32.2	10.6	6.9%	-32.20 [-52.98, -11.42]
**Subtotal (95% CI)**			**35.9%**	**-36.10 [-44.37, -27.84]**
Heterogeneity: Tau^2 = 0.00; Chi^2 = 4.81, df = 5 (P = 0.44); I^2 = 0%				
Test for overall effect: Z = 8.56 (P < 0.00001)				
**1.1.2 Prescription Drug Monitoring Program or State Law**				
Borstein et al,(72) 2019	3.1	16.4	5.0%	3.10 [-29.04, 35.24]
Duppong et al,(28) 2019	-48.1	8	7.7%	-48.10 [-63.78, -32.42]
Liu et al,(73) 2020	-0.12	4.5	8.8%	-0.12 [-8.94, 8.70]
Suffoletto et al,(26) 2018	-17.4	5.4	8.5%	-17.40 [-27.98, -6.82]
Sun et al,(70) 2017	-8.5	11.8	6.5%	-8.50 [-31.63, 14.63]
Sun et al,(71) 2018	-3.6	8.8	7.5%	-3.60 [-20.85, 13.65]
**Subtotal (95% CI)**			**44.0%**	**-13.43 [-28.70, 1.85]**
Heterogeneity: Tau^2 = 280.06; Chi^2 = 30.17, df = 5 (P < 0.0001); I^2 = 83%				
Test for overall effect: Z = 1.72 (P = 0.08)				
**1.1.3 Clinicians Peer Comparison**				
Andereck et al,(88) 2019	-17.2	9.2	7.3%	-17.20 [-35.23, 0.83]
Boyle et al,(89) 2019	-110	46	1.2%	-110.00 [-200.16, -19.84]
Dieujuste et al,(90) 2020	-19.1	18.5	4.5%	-19.10 [-55.36, 17.16]
Guarisco et al,(86) 2018	-38.2	23.9	3.3%	-38.20 [-85.04, 8.64]
Meisenberg et al,(87) 2018	-78.8	21.2	3.8%	-78.80 [-120.35, -37.25]
**Subtotal (95% CI)**			**20.1%**	**-41.27 [-69.98, -12.57]**
Heterogeneity: Tau^2 = 606.99; Chi^2 = 10.70, df = 4 (P = 0.03); I^2 = 63%				
Test for overall effect: Z = 2.82 (P = 0.005)				
**Total (95% CI)**			**100.0%**	**-27.19 [-37.77, -16.61]**
Heterogeneity: Tau^2 = 312.58; Chi^2 = 70.06, df = 16 (P < 0.00001); I^2 = 77%				
Test for overall effect: Z = 5.04 (P < 0.00001)				
Test for subgroup differences: Chi^2 = 7.02, df = 2 (P = 0.03), I^2 = 71.5%				

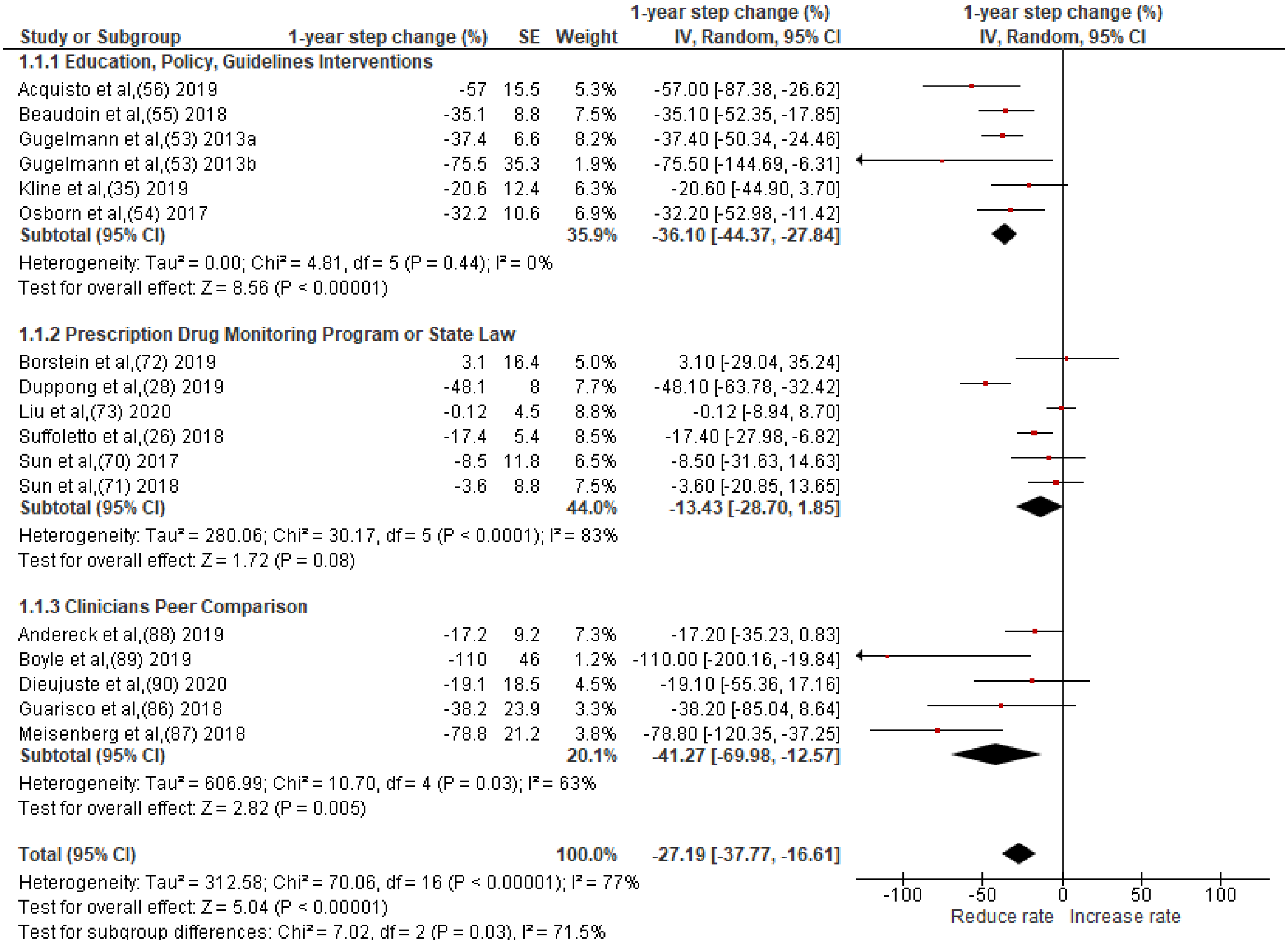

© 2022 Daoust R et al. *JAMA Network Open*.

eFigure 7. Forest Plot of the 1-y Step Change in Prescribed Opioid Quantity for ITS Studies

Study or Subgroup	1-year step change (%)	SE	Weight	1-year step change (%) IV, Random, 95% CI	Year
**2.1.1 Education, Policy, Guidelines interventions**					
Osborn et al,(54) 2017	-10.9	2.8	11.9%	-10.90 [-16.39, -5.41]	2017
Kline et al,(35) 2019	-26.5	11.4	6.9%	-26.50 [-48.84, -4.16]	2019
**Subtotal (95% CI)**			**18.8%**	**-14.79 [-28.01, -1.56]**	
Heterogeneity: Tau² = 52.78; Chi² = 1.77, df = 1 (P = 0.18); I² = 43%					
Test for overall effect: Z = 2.19 (P = 0.03)					
**2.1.2 Prescription Drug Monitoring Program or State Law**					
Sun et al,(70) 2017	-6.8	7.3	9.4%	-6.80 [-21.11, 7.51]	2017
Suffoletto et al,(26) 2018	7.9	4	11.3%	7.90 [0.06, 15.74]	2018
Sun et al,(71) 2018	4	0.77	12.4%	4.00 [2.49, 5.51]	2018
**Subtotal (95% CI)**			**33.1%**	**3.94 [-0.60, 8.48]**	
Heterogeneity: Tau² = 7.28; Chi² = 3.14, df = 2 (P = 0.21); I² = 36%					
Test for overall effect: Z = 1.70 (P = 0.09)					
**2.1.3 Electronic Medical Record Quantity Changes**					
Delgado et al,(36) 2018a	21.4	14.3	5.5%	21.40 [-6.63, 49.43]	2018
Delgado et al,(36) 2018b	-5.4	8.5	8.6%	-5.40 [-22.06, 11.26]	2018
Slovis et al,(94) 2018	-62.8	9.7	7.9%	-62.80 [-81.81, -43.79]	2018
Beauchamps et al,(95) 2020	-12.4	11.4	6.9%	-12.40 [-34.74, 9.94]	2020
Villwock et al,(96) 2020	12	10.8	7.3%	12.00 [-9.17, 33.17]	2020
Carlson et al,(97) 2021	-14.6	2.8	11.9%	-14.60 [-20.09, -9.11]	2020
**Subtotal (95% CI)**			**48.1%**	**-11.45 [-29.92, 7.01]**	
Heterogeneity: Tau² = 434.85; Chi² = 38.30, df = 5 (P < 0.00001); I² = 87%					
Test for overall effect: Z = 1.22 (P = 0.22)					
**Total (95% CI)**			**100.0%**	**-8.34 [-17.16, 0.49]**	
Heterogeneity: Tau² = 162.82; Chi² = 122.07, df = 10 (P < 0.00001); I² = 92%					
Test for overall effect: Z = 1.85 (P = 0.06)					
Test for subgroup differences: Chi² = 8.82, df = 2 (P = 0.01), I² = 77.3%					

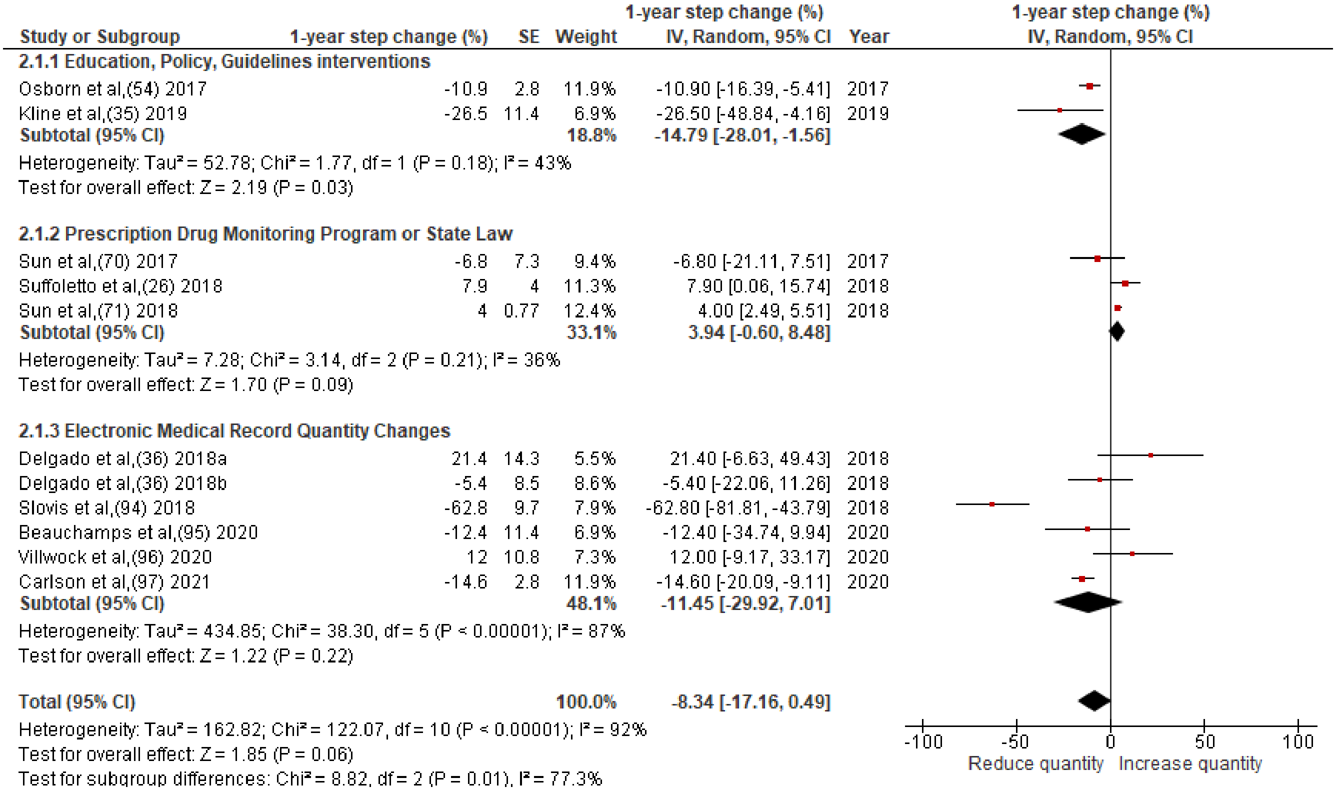

© 2022 Daoust R et al. *JAMA Network Open*.

eFigure 8. Forest Plot of the 6-mo Step Change in Opioid Prescription Rate for ITS Studies Excluding Studies at High Risk of Bias

Study or Subgroup	6-month step change (%)	SE	Weight	6-month step change (%) IV, Random, 95% CI
**1.1.1 Education, Policy, Guidelines Interventions**				
Acquisto et al,(56) 2019	-40.9	9.1	9.9%	-40.90 [-58.74, -23.06]
Beaudoin et al,(55) 2018	-32	5.8	15.0%	-32.00 [-43.37, -20.63]
Gugelmann et al,(53) 2013a	-34.7	5.4	15.8%	-34.70 [-45.28, -24.12]
Gugelmann et al,(53) 2013b	-46.3	21.2	2.8%	-46.30 [-87.85, -4.75]
Kline et al,(35) 2019	-20.6	12.4	6.6%	-20.60 [-44.90, 3.70]
Osborn et al,(54) 2017	-29.2	9.5	9.4%	-29.20 [-47.82, -10.58]
**Subtotal (95% CI)**			**59.5%**	**-33.32 [-39.65, -26.99]**
Heterogeneity: Tau² = 0.00; Chi² = 2.43, df = 5 (P = 0.79); I² = 0%				
Test for overall effect: Z = 10.32 (P < 0.00001)				
**1.1.2 Prescription Drug Monitoring Program or State Law**				
Borstein et al,(72) 2019	-3.9	12.3		Not estimable
Duppong et al,(28) 2019	-32.3	5.4		Not estimable
Liu et al,(73) 2020	0.41	4		Not estimable
Suffoletto et al,(26) 2018	-16.6	4.8	16.9%	-16.60 [-26.01, -7.19]
Sun et al,(70) 2017	-7.3	6.9		Not estimable
Sun et al,(71) 2018	-4	7.2		Not estimable
**Subtotal (95% CI)**			**16.9%**	**-16.60 [-26.01, -7.19]**
Heterogeneity: Not applicable				
Test for overall effect: Z = 3.46 (P = 0.0005)				
**1.1.3 Clinicians Peer Comparison**				
Andereck et al,(88) 2019	-17.2	9.2	9.8%	-17.20 [-35.23, 0.83]
Boyle et al,(89) 2019	-66.1	27.4	1.8%	-66.10 [-119.80, -12.40]
Dieujuste et al,(90) 2020	-13.5	13.7	5.7%	-13.50 [-40.35, 13.35]
Guarisco et al,(86) 2018	-19.5	13		Not estimable
Meisenberg et al,(87) 2018	-50.5	12.7	6.4%	-50.50 [-75.39, -25.61]
**Subtotal (95% CI)**			**23.6%**	**-31.37 [-53.15, -9.60]**
Heterogeneity: Tau² = 280.95; Chi² = 7.56, df = 3 (P = 0.06); I² = 60%				
Test for overall effect: Z = 2.82 (P = 0.005)				
**Total (95% CI)**			**100.0%**	**-29.36 [-36.75, -21.97]**
Heterogeneity: Tau² = 61.09; Chi² = 18.35, df = 10 (P = 0.05); I² = 46%				
Test for overall effect: Z = 7.78 (P < 0.00001)				
Test for subgroup differences: Chi² = 8.44, df = 2 (P = 0.01), I² = 76.3%				

6-month step change (%)
IV, Random, 95% CI

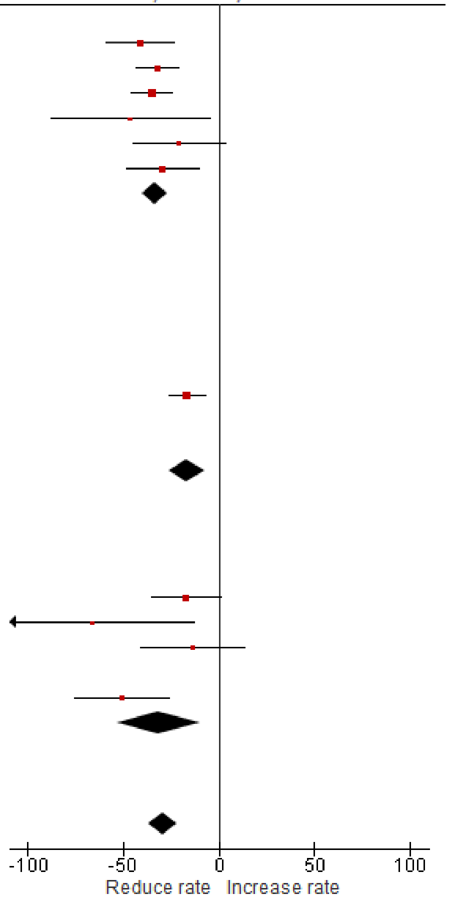

© 2022 Daoust R et al. *JAMA Network Open*.

eFigure 9. Forest Plot of the 6-mo Step Change in Prescribed Opioid Quantity for ITS Studies Excluding Studies at High Risk of Bias

Study or Subgroup	6-month step change (%)	SE	Weight	6-month step change (%) IV, Random, 95% CI
**2.1.1 Education, Policy, Guidelines interventions**				
Kline et al,(35) 2019	-26.5	11.4	24.4%	-26.50 [-48.84, -4.16]
Osborn et al,(54) 2017	-13.4	3.2	38.0%	-13.40 [-19.67, -7.13]
**Subtotal (95% CI)**			**62.4%**	**-15.38 [-24.58, -6.18]**
Heterogeneity: Tau² = 15.70; Chi² = 1.22, df = 1 (P = 0.27); I² = 18%				
Test for overall effect: Z = 3.28 (P = 0.001)				
**2.1.2 Prescription Drug Monitoring Program or State Law**				
Suffoletto et al,(26) 2018	7.2	3.5	37.6%	7.20 [0.34, 14.06]
Sun et al,(70) 2017	-5.7	11.1		Not estimable
Sun et al,(71) 2018	3.5	0.64		Not estimable
**Subtotal (95% CI)**			**37.6%**	**7.20 [0.34, 14.06]**
Heterogeneity: Not applicable				
Test for overall effect: Z = 2.06 (P = 0.04)				
**2.1.3 Electronic Medical Record Quantity Changes**				
Beauchamps et al,(95) 2020	-12.4	11.4		Not estimable
Carlson et al,(97) 2021	-14.3	2.4		Not estimable
Delgado et al,(36) 2018a	21.4	14.3		Not estimable
Delgado et al,(36) 2018b	-5.4	8.5		Not estimable
Slovis et al,94 2018	-62.8	9.7		Not estimable
Villwock et al,(96) 2020	9.1	8.7		Not estimable
**Subtotal (95% CI)**				**Not estimable**
Heterogeneity: Not applicable				
Test for overall effect: Not applicable				
**Total (95% CI)**			**100.0%**	**-8.85 [-26.61, 8.90]**
Heterogeneity: Tau² = 205.92; Chi² = 22.60, df = 2 (P < 0.0001); I² = 91%				
Test for overall effect: Z = 0.98 (P = 0.33)				
Test for subgroup differences: Chi² = 14.88, df = 1 (P = 0.0001), I² = 93.3%				

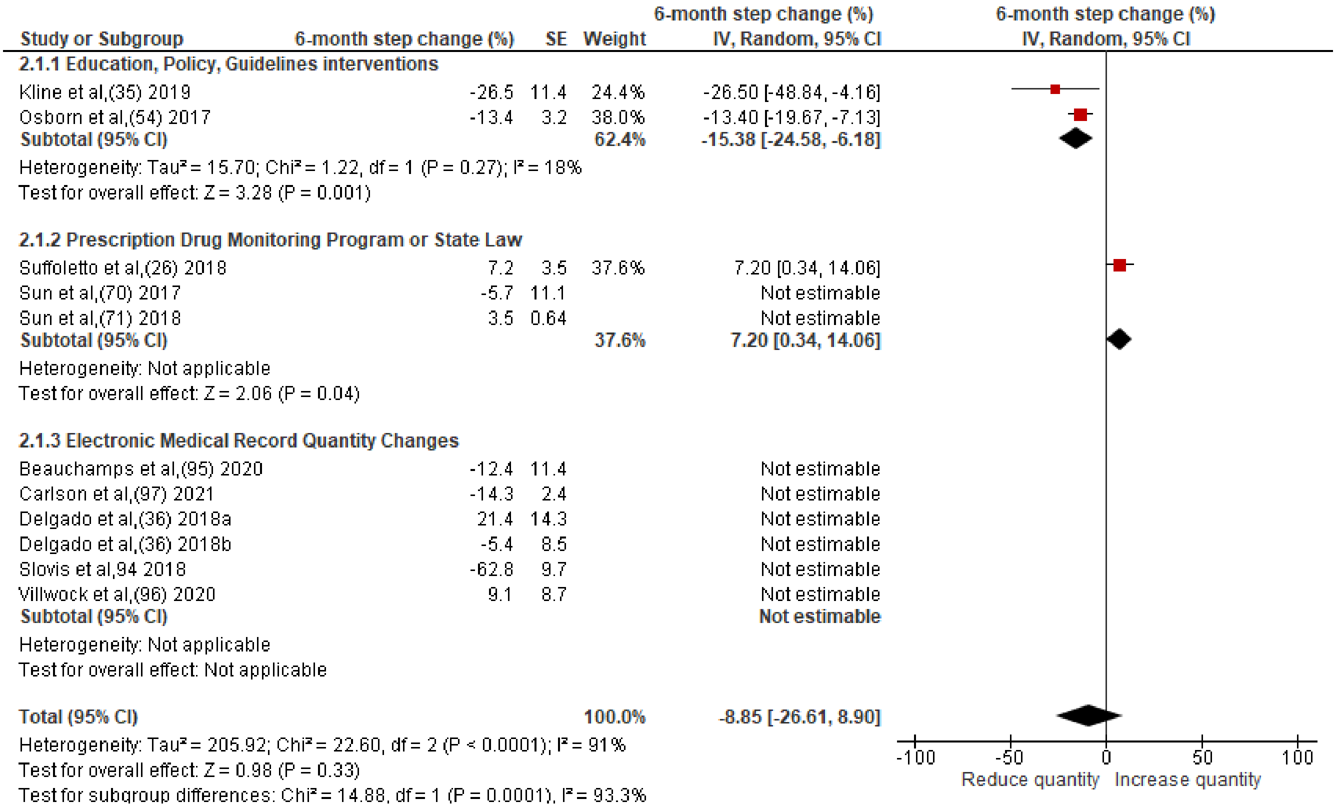

© 2022 Daoust R et al. *JAMA Network Open*.

eFigure 10. Funnel Plot With Pseudo 95% CIs of the 6-mo Step Change in the Opioid Prescription Rate for ITS Studies

(Egger regression intercept: -1.65; SE: 1.14; p=0.17). EPG: Education, Policy, or Guidelines evaluation; PDMP: Prescription Drug Monitoring Program or State Law; CPC: Clinician Peer Comparison opioid prescribing

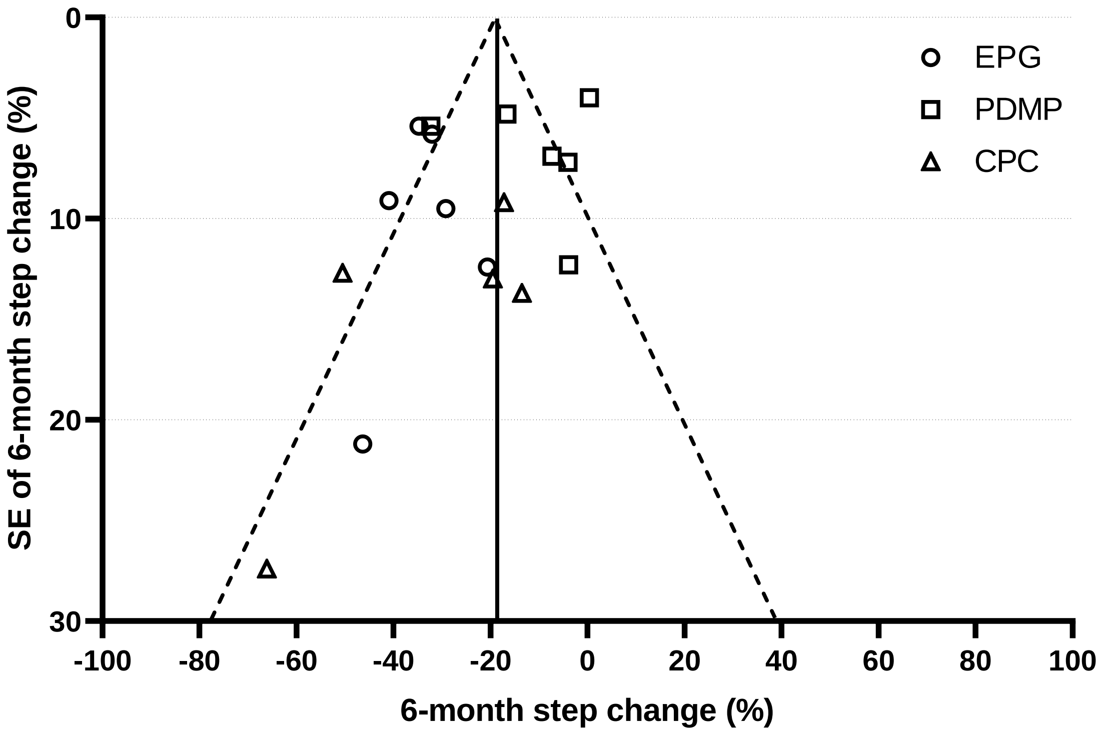

© 2022 Daoust R et al. *JAMA Network Open*.

eFigure 11. Funnel Plot With Pseudo 95% CIs of the 6-mo Step Change in the Opioid Prescription Rate for RCT, Preintervention-Postintervention, and Cohort Studies

(Egger regression intercept: 0.24; SE: 3.4; p=0.94). EPG: Education, Policy, or Guidelines evaluation; PDMP: Prescription Drug Monitoring Program or State Law; CPC: Clinician Peer Comparison opioid prescribing; PT: Physical Therapy; EMR_QC: Electronic Medical Record Quantity Changes

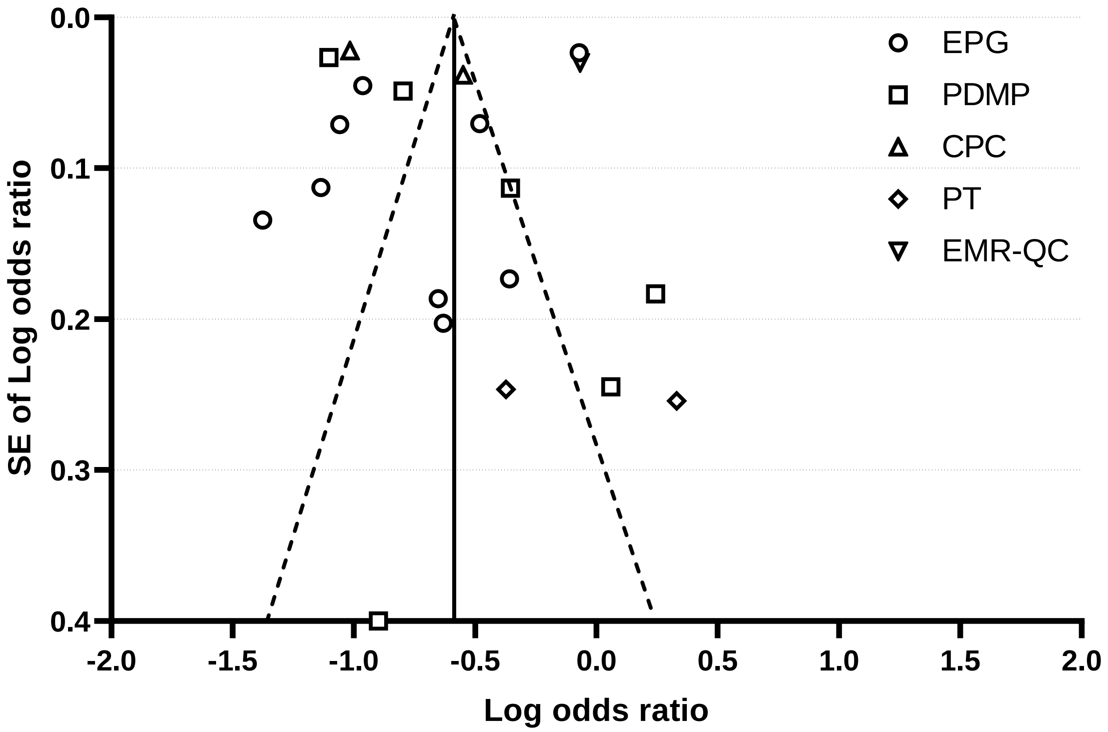

© 2022 Daoust R et al. *JAMA Network Open*.

eFigure 12. Funnel Plot With Pseudo 95% CIs of the 6-mo Step Change in the Opioid Prescription Rate in the Prescribed Opioid Quantity for ITS Studies

(Egger regression intercept: -2.22; SE: 1.22; p=0.10). EPG: Education, Policy, or Guidelines evaluation; PDMP: Prescription Drug Monitoring Program or State Law; EMR_QC: Electronic Medical Record Quantity Changes

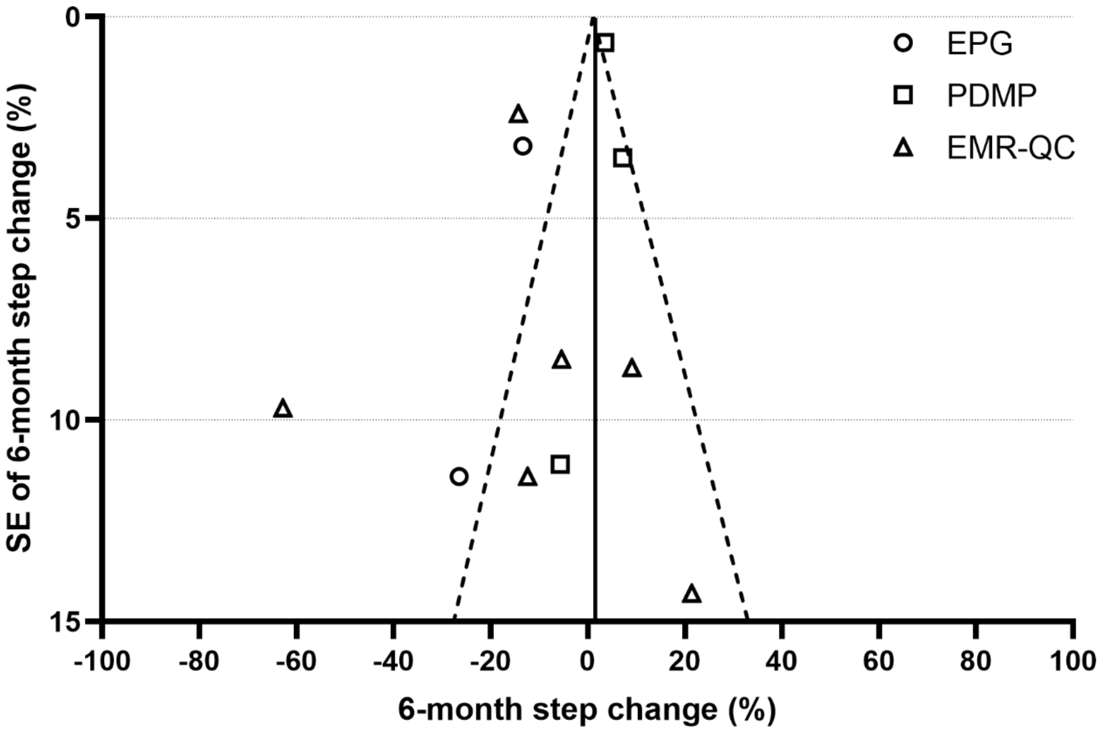

© 2022 Daoust R et al. *JAMA Network Open*.

eFigure 13. Funnel Plot With Pseudo 95% CIs of the 6-mo Step Change in the Prescribed Opioid Quantity for RCT and Preintervention-Postintervention Studies

(Egger regression intercept: 7.08; SE: 5.92; p=0.26). EPG: Education, Policy, or Guidelines evaluation; PDMP: Prescription Drug Monitoring Program or State Law; CPC: Clinician Peer Comparison opioid prescribing; EMR_QC: Electronic Medical Record Quantity Changes

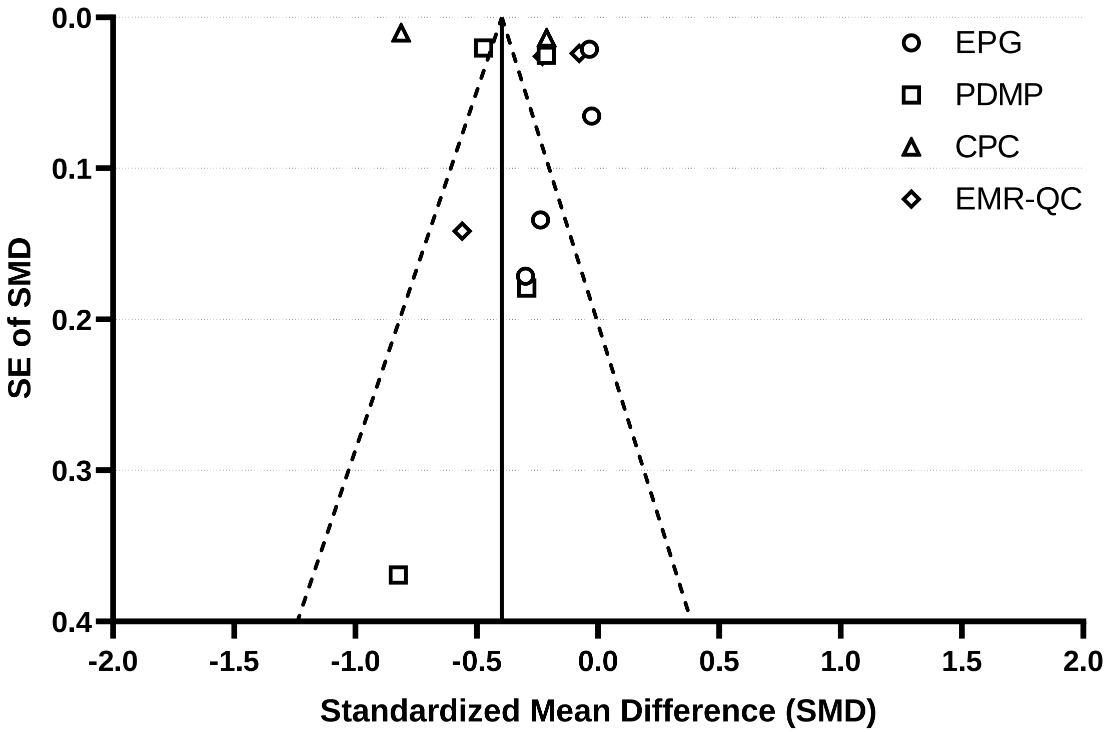

© 2022 Daoust R et al. *JAMA Network Open*.

eReferences

1. Koo TK, Li MY. A guideline of selecting and reporting intraclass correlation coefficients for reliability research. *J Chiropr Med*. 2016;15(2):155-163.
2. Cochrane Effective Practice and Organisation of Care (EPOC). Interrupted time series (ITS) analyses. EPOC resources for review authors, 2017. Accessed December 15, 2020. https://epoc.cochrane.org/resources/epoc-specific-resources-review-authors.
3. Garcia-Elorrio E, Rowe SY, Teijeiro ME, Ciapponi A, Rowe AK. The effectiveness of the quality improvement collaborative strategy in low- and middle-income countries: a systematic review and meta-analysis. *PLoS One*. 2019;14(10):e0221919.
